# A new endophytic fungus CJAN1179 isolated from the Cholistan desert promotes lateral root growth in *Arabidopsis* and produces IAA through tryptophan-dependent pathway

**DOI:** 10.1007/s00203-022-02768-2

**Published:** 2022-02-17

**Authors:** Adeela Naureen, Faiz-ul H. Nasim, Muhammad S. Choudhary, Muhammad Ashraf, Florian M. W. Grundler, A. Sylvia S. Schleker

**Affiliations:** 1 Chemistry Department, The Islamia University of Bahawalpur, Bahawalpur, 63000 Pakistan; 2 Department of Botany, The Islamia University of Bahawalpur, Bahawalpur, 63000 Pakistan; 3 INRES, Molecular Phytomedicine, Rheinische Friedrich-Wilhelms-University Bonn, Karlrobert-Kreiten-Str. 13, 53115 Bonn, Germany; 4 Institute of Biochemistry, Biotechnology and Bioinformatics, The Islamia University of Bahawalpur, Baghdad Ul Jadeed Campus, Bahawalpur, 63000 Pakistan

**Keywords:** Plant growth promotion, Cholistan desert, *Cymbopogon jwarancusa*, Root-associated fungi, Tryptophan, Auxin production

## Abstract

Fungi, important for growth of plants in arid lands, are expected to be involved in novel biochemical activities during fungal–plant interactions. We isolated 150 fungi associated with rhizosphere and root endosphere of two perennial grasses, *Cymbopogon jwarancusa* and *Panicum antidotale*, from Cholistan desert. The isolates were screened for their impact on plant growth and development using *Arabidopsis thaliana (Col-0)* as a model system. A root-endophytic fungus CJAN1179 from *C. jwarancusa* showed the highest plant growth-promoting effects. The most remarkable was enhanced number of lateral roots (3.1-fold). CJAN1179 produced indole-3-acetic acid (IAA) particularly in the presence of tryptophan. ITS sequence and phylogenetic analysis characterisation suggested the fungus to be a new species within *Sordariomycetidae.* CJAN1179 appears to promote plant growth by secreting IAA using tryptophan as a precursor. This fungus can be further explored for its suitability to promote growth of commercially important crops, particularly in arid regions.

**Supplementary Information:**

The online version contains supplementary material available at 10.1007/s00203-022-02768-2.

## Introduction

Activities of a great variety of microorganisms including rhizospheric and endophytic fungi living in association with plants influence plant phenotype and development (Rodriguez et al. 2009; Lugtenberg et al. 2013). These plant-associated microorganisms contribute to plant survival (Rodriguez et al. 2004; Ortíz-Castro et al. 2009). Plants growing and surviving in habitats with extreme climatic conditions such as arid soils develop functional diversity and the root-associated microbial populations must be contributing their important role here (Zuo et al. 2015). Deserts are considered to be among the habitats where plants are exposed to extreme environmental conditions such as drought, high temperature, extreme pH and salinity. Root-associated microbial communities of desert plants have been suggested as an important factor responsible for adaptation of desert plants to the harsh desert environment (Bhatnagar and Bhatnagar 2005; Cherif et al. 2015; Makhalanyane et al. 2015). It is, therefore, of high interest to explore the diversity and role of microorganisms originating from desert plants for plant growth and survival. The Cholistan desert located in the southern part of Bahawalpur in Punjab, Pakistan is a largely unexplored habitat in this respect (Rafay et al. 2013). Its flora is composed of xerophytic species that have adapted to the desert conditions (Arshad et al. 2008; Hameed et al. 2011). Unique microbial communities producing novel metabolites involved in beneficial fungus–plant interactions are expected to reside in association with the roots of plants growing in this desert.

Fungi are a group of diverse organisms with multiple metabolic activities and a large group of endophytic fungi establishes mycorrhizal or non-mycorrhizal associations with the plant roots (Rodriguez et al. 2009). Endophytic fungi have been reported to mediate a number of beneficial roles inside plants (Rodriguez et al. 2004; Gond et al. 2010; Khan et al. 2012; Zhou et al. 2014) including involvement of endophytes in plant nutrient uptake and the production of secondary metabolites to stimulate plant growth (Waqas et al. 2012). However, the mechanistic biochemical details of endophyte–plant associations are often not clearly understood (Dovana et al. 2015).

When fungi and plant roots sense the presence of each other in rhizosphere, they produce certain metabolites to initiate a mutualistic fungus–plant interaction that is well organised and bidirectional. Endophytes absorb required nutrients from plant roots to produce metabolites that directly influence plant growth promotion or indirectly by initiating plant-signalling mechanisms leading to the production of growth-promoting molecules, especially phytohormones (Ortíz-Castro et al. 2009). A number of plant beneficial fungi have been reported to produce phytohormones (Aloni et al. 2006; Hamayun et al. 2010). Microbial phytohormones, e.g. auxins, cytokinins and gibberellins are either involved in plant growth promotion or help plant cope with biotic or abiotic stress. Thus, root-associated plant beneficial fungi can be explored as an environment-friendly tool to promote plant growth and ensure high productivity (Rodriguez et al. 2009; Mei and Flinn 2010).

Towards exploring the diversity and beneficial properties of cultivable desert plant root-associated fungi, we collected 150 fungal isolates from the rhizosphere and endosphere of *Cymbopogon jwarancusa* and *Panicum antidotale*, two perennial grasses commonly growing in the Cholistan desert. We screened these fungal isolates for their impact on growth and development using *A. thaliana* as a model. The knowledge of plant root-associated fungi and their beneficial roles during interaction with *A. thaliana* has provided insights into plant–microbe associations and is expected to identify microorganisms suitable for use in sustainable agricultural management strategies.

## Materials and methods

### Sample collection

Roots with attached soil from three ecotypes of *P. antidotale* P1 (accession # PA-YZ-PL14-18), P4 (accession # PA-YZ-PL02-37) and P10 (accession # PA-YZ-PLH-28) and two ecotypes of *C. jwarancusa* C10 (accession # CH-MW-PL10-13) and C12 (accession # CH-NT-PL12-16) were collected on 14 March 2013 in sterile plastic jars from the Cholistan Institute of Desert Studies (CIDS) located between 27°42ʹ and 29°45ʹ N latitude and 69°52ʹ and 75°24ʹ E longitude, the Islamia University of Bahawalpur (IUB), Pakistan. Collected samples were stored in a cool and dark place until further processing.

### Isolation of fungi from rhizospheric soils

Rhizospheric soil of each sample was suspended in 30 ml sterile Ringer solution. 100 µl of the optimal dilution of this solution suitable to obtain separated fungal colonies, was spread on Sabouraud Agar (SA) (pH = 6.8) plates including Streptomycin and Penicillin (100 and 50 mg/L, respectively) to inhibit bacterial growth (Sun et al. 2009). The plates were incubated in inverted position at 37 °C until fungal colonies appeared. As negative control 100 µl sterile Ringer solution was spread on SA plates in parallel. A liquid culture of each fungal isolate was grown in Sabouraud’s Broth, (SB) pH = 6.8 at 37 °C and 250 rpm. Fungal isolates were initially characterised on the basis of morphology. Duplication was avoided. The fungal spores were stored in 50% SB glycerol at − 70 °C.

### Isolation of root-endophytic fungi

To isolate root endophytes, roots were surface sterilised using an earlier described method (Ahmed et al. 2012) with a few modifications. All attached soil was thoroughly washed off using tap water. The roots were then rinsed stepwise with 70% ethanol for 30 s, 0.01% HgCl_2_ for 5 min, 0.5% NaOCl for 3 min followed by washing with sterile water to remove chemicals. These roots were dried between folds of sterile filter paper and cut into small pieces of about 8 mm that were placed on SA plates for fungal isolation. The plates were incubated as describes above. The water from last wash before drying the roots was plated on SA plates as negative control (Fouda et al. 2015). Spores of isolated endophytic fungi were stored as stated earlier.

### Screening fungal isolates for their impact on plant development

The impact of 150 fungal isolates on growth and development of wild-type *Arabidopsis thaliana Columbia-0 (Col-0)* was tested in fungus–plant interaction assays performed as follows. Freshly grown purified fungal colonies on SA plates were used for fungus–plant interaction assays.

*A. thaliana* seeds were sterilised through slightly modified standard method (Sijmons et al. 1991; Bohlmann and Wieczorek 2015). In brief, seeds were placed in 0.6% NaOCl for 5 min. The supernatant was removed and the seeds were soaked in 70% ethanol for 5 min followed by washing them 5 times with sterile water and drying in between the layers of sterile filter paper. Surface sterilised dried seeds were stored at 4 °C until use. For the interaction assays, two sterile seeds were placed 4 cm apart and at 1.5 cm from the upper edge of the plate on 2% Knop agar plates that were incubated in tilted position in a growth chamber for 6 days (23 ± 1 °C, 16:8, light/dark, photoperiod, light intensity of 150 µmol m^−2^ s^−2^ of PAR). To determine the impact of the fungal isolates on plant growth and to allow proper comparison, all the plants used for the interaction assay had to fulfil the following criteria on day 6: primary root length of almost 2.5 cm, rosette diameter of about 0.8 cm and 4 leaves.

A fungal plug of 5 mm diameter was placed on the plate at 5 cm distance from the root end of the 6-day-old plants. These plates along with control plates (plants without fungal exposure) were incubated in a growth chamber as described above.

Parameters related to plant growth including primary root length (cm), number of lateral roots, root hairs, number of leaves, rosette diameter (cm), leaf colour, fresh shoot weight (g), flower stalk length (cm) and flower blossom status were recorded during the assay at specific intervals. The first measurement of these parameters was done at the time of fungal inoculation, 0 days’ post-inoculation (dpi). Subsequent measurements were performed at equal intervals of 4 days up to 16 dpi. Among all parameters, root hairs, leaf colour and flower blossom were qualitative parameters and determined visually. The initial screening of all 150 fungal isolates for their impact on plant development was done with 2 plants per fungal isolate on Knop agar plate.

### Detailed analysis of the impact of selected fungal isolates on plant development

Out of 150 fungi, a root endophyte CJAN1179 obtained from *C. jwarancusa* possessed the most remarkable beneficial effect on plant root exhibited as highly enhanced number of lateral roots and little effect on shoot growth. Isolates PAAN1181 and PAAN1135 obtained from *P. antidotale* exhibited the worst negative effects and almost no effect on plant growth, respectively.

To validate the observed plant growth-promoting properties of CJAN1179, a detailed analysis was performed using PAAN1181 and PAAN1135 as controls in the presence or in the absence of CJAN1179 for comparison. The fungus–plant interaction assay was done in 3 biological replicates with 10 technical replicates each (30 plants each for each variant in total). The assay was done as described above with the exception that besides fungal plug inoculation (FPI), a second fungus–plant interaction assay setup using fungal culture filtrates was performed. FPI was done using 6-day-old plants fulfilling the criteria described above (Ortíz-Castro et al. 2009). Fungal culture filtrate inoculation (FCFI) was done with 9-day-old plants with the following growth status: root length around 5 cm, number of lateral roots almost 10, number of leaves 6 and rosette diameter almost 2 cm. At this time point, the roots attain enough root mass to absorb culture filtrate (Daneshkhah et al. 2013).

For the FCFI assay, liquid cultures of the three fungi were grown in SB at 37 ˚C on a rotary incubator at 250 rpm for 14 days. Each culture was filtered through 0.2 µm membrane syringe filter. Fungal culture filtrates and control (150 µl) were directly applied onto the plant roots and left for half an hour allowing plant roots to absorb the filtrate. The treated plants were incubated in a growth chamber. The parameters to document plant development were recorded as described above.

### Statistical analysis

The obtained data were analysed using SPSS 15. Plant growth parameters were taken as dependent variable. Fungal inoculations (FPIs/FCFIs) and dpi were taken as fixed factors or independent variables. Some plant growth parameters were analysed with two fixed factors (fungal inoculations and dpi) through descriptive statistics and significance (*P* < 0.05) using “Univariant Analysis of Variance”. Parameters measured at only 16 dpi were analysed with only one fixed factor (fungal inoculations) using “One way ANOVA”. Least significant difference (LSD) between control plant and inoculated plant growth parameters were calculated based on observed means through “Post Hoc Tests”. The mean differences were categorised as significant on the basis of the size of effect as: very significant P** (*P* ≤ 0.01), significant P* (*P* ≤ 0.05) and non-significant *P*^NS^ (*P* > 0.05). All quantitative plant growth parameters were statistically analysed. Root hairs, colour of leaves and flower blossom were not statistically evaluated because these are qualitative parameters.

### Detection of auxin (IAA) production

Salkowski colorimetric assay was used to determine whether CJAN1179 produces indole-3-acetic acid (IAA), a predominant naturally occurring auxin (Glickmann and Dessaux 1995). This assay is the most recommended method for the detection of IAA production in microbial cultures (Sarwar and Kremer 1995; Mohite 2013; Rehman et al. 2015).

A standard curve (absorbance vs. concentrations) was prepared by mixing different amounts of a 0.01% IAA solution (0 µl, 5 µl, …30 µl), 100 µl Salkowski reagent and ddH_2_O up to a total volume of 250 µl in triplicate. After 20 min of incubation at 26 °C, the OD was measured at 530 nm using a plate reader.

The three fungi (CJAN1179, PAAN1135 and PAAN1181) were cultured in SB with and without 1% L-tryptophan using the same culture conditions. The reaction mixture (250 µl) containing different pre-determined volumes of fungal culture filtrates (CFs), viz., CJAN1179, PAAN1135 and PAAN1181 CF with and without tryptophan, and IAA controls were prepared in triplicate and handled as described above. IAA concentration of each sample was calculated using the standard curve.

### Identification of beneficial fungus CJAN1179

#### Molecular characterisation

Genomic DNA of CJAN1179 was isolated with some modifications in already reported method (Liu et al. 2000). 25 mg of freshly grown colony of CJAN1179 SA was taken into a 2-ml tube containing stainless steel beads of 5 mm diameter and 500 μl lysis buffer (400 mM Tris–HCl, 60 mM EDTA, 150 mM NaCl and 1% SDS, pH = 8.0). The tube was left at room temperature for 5 min. To this solution, 150 µl of potassium acetate solution (60 ml 5 M potassium acetate, 11.5 ml glacial acetic acid, 28.5 ml ddH_2_O, pH 4.8) was added and the tube was placed in Tissue Lyser for lysis of fungal colony at 40 Hz for 10 min. The tube was then placed at − 30 °C for 20 min and centrifuged at 17,135 × *g* for 5 min. Supernatant from the tube was transferred to another tube and extracted twice with equal volumes of chloroform: isoamyl alcohol (24:1) before centrifugation. The supernatant was then mixed with an equal volume of isopropanol. The tube was placed at 4 °C for 1 h and centrifuged at 17,135 × *g* for 5 min. The DNA pellet was washed 3 times with 300 µl 70% ethanol, air dried and dissolved in 50 µl of 1x Tris EDTA. Integrity of the extracted DNA was verified by agarose gel electrophoresis.

For amplification, 10 ng fungal DNA was used as template in a standard PCR reaction containing 10 mM 1X PCR buffer, 1.5 mM MgCl_2_, 100 mM of each of the four dNTPs, 1.25 U Taq DNA polymerase and 10 pmol of each of the two primers ITS1 and ITS4, forward and reverse primers, respectively. Amplification conditions were as follows: initial denaturation at 94 °C for 5 min, 30 cycles of 94 °C for 30 s, 1 min at 50 °C and 1 min at 72 °C, followed by final extension at 72 °C for 10 min. Expected size of the PCR product was verified by agarose gel electrophoresis. The DNA fragment was cleaned, purified and sent to GATC Biotech, Cologne Germany for sequencing.

#### Phylogenetic analysis

For phylogenetic lineage determination of CJAN1179, the route of analysis followed was adopted from a published description with some modifications (Nasim et al. 2018).

- Multiple sequence alignment of ITS sequences from query and selected fungal species: NCBI BLAST was used to reveal the number of maximum hits for the query sequence and to retrieve the most similar sequences for construction of the phylogenetic tree (Altschul et al. 1997). Genious bioinformatics software was used to perform multiple sequence alignment of our query along with selected sequences (Kearse et al. 2012). In multiple sequence alignment, truncated sequences were deleted and longer sequences were shortened to make them all equal in length.
- Molecular phylogenetic analysis of ITS for query along with closely related fungal species: Neighbour-joining method (Saitou and Nei 1987) was used to infer the evolutionary history. Evolutionary history of the taxa analysed was represented using the bootstrap consensus tree inferred from 100 replicates. Branches corresponding to partitions reproduced in less than 50% bootstrap replicates were collapsed. The percentage of replicate trees in which the associated taxa clustered together in the bootstrap test (100 replicates) are shown next to the branches (Felsenstein 2005). The tree was drawn to scale, with branch lengths in the same units as those of the evolutionary distances used to infer the phylogenetic tree. P-distance method (Nei and Kumar 2000) was used to compute the evolutionary distances which are in the units of the number of base differences per site. The analysis involved 38 nucleotide sequences. All positions containing gaps and missing data were eliminated. A total of 429 positions in the final dataset were available in this analysis. MEGA5 (Tamura et al. 2011) was used to conduct evolutionary analyses.

#### Morphological characterisation

After phylogenetic analysis of the fungal sequence on the basis of ITS regions using molecular and phylogenetic tools, the potential fungus CJAN1179 was grown both on SA plates and in SB medium for possible macroscopic studies, i.e. shape and colour of fungal colony and spores to establish further placement of fungus in taxonomic lineage. Detailed microscopic studies were not performed as molecular tools give more precise taxonomic information and are preferred over conventional morphological parameters for identification of the fungus (Tarbell 2008) and ITS region is considered the most suitable DNA barcode for fungi (Schoch et al. 2012).

## Results

### Isolation of fungi

In total, 150 morphologically different fungi were isolated. Among those, 79 (48 rhizospheric and 31 root endophytes) fungi were isolated from three ecotypes of *P. antidotale*, whereas 71 (56 rhizospheric and 15 root endophytes) fungi were isolated from two ecotypes of *C. jwarancusa.*

### Root endophyte CJAN1179 is a plant growth-promoting fungus

**Fig. 1:**
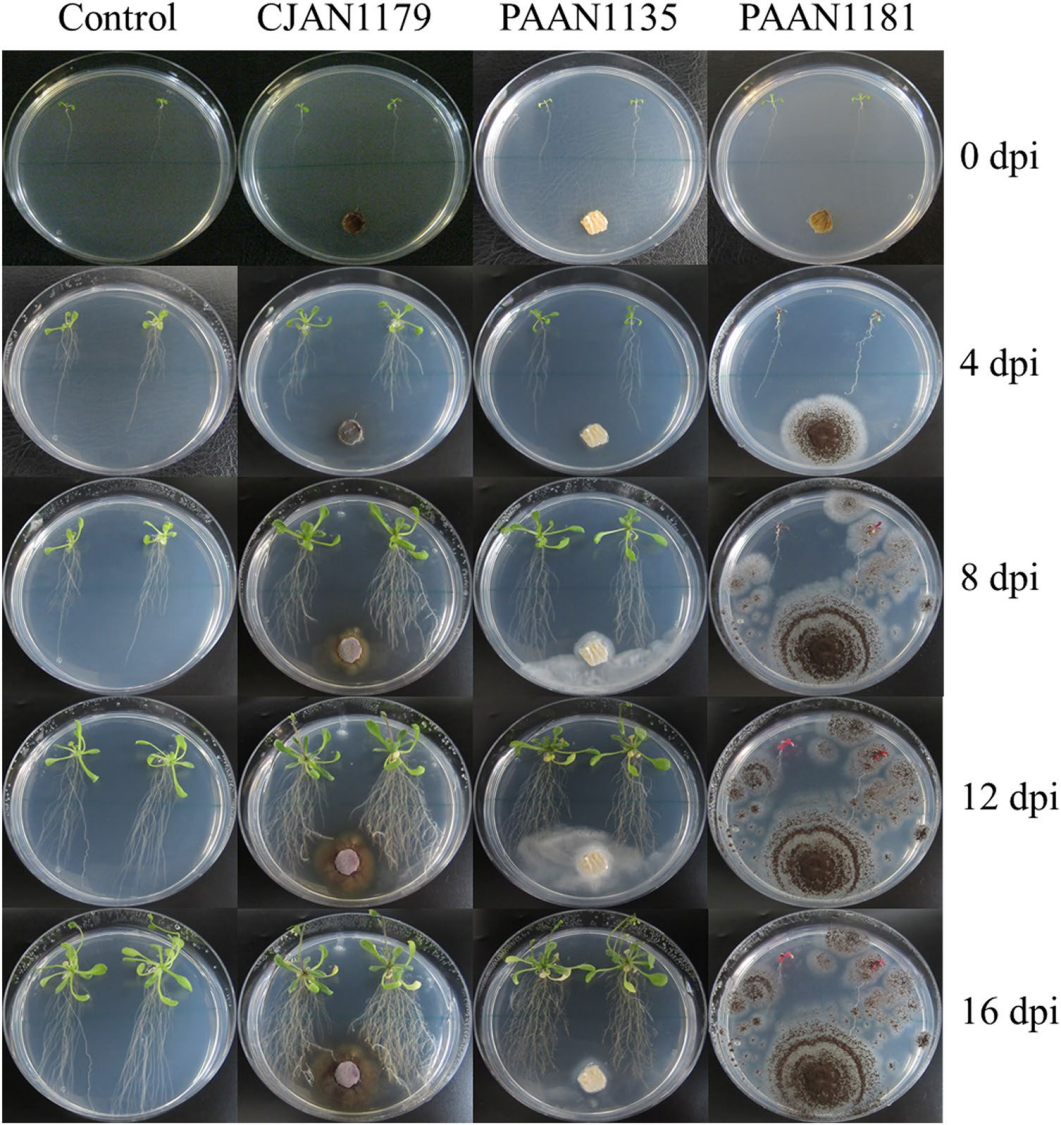
Excessive lateral root formation induced by CJAN1179 in *A. thaliana* as compared to unexposed control plants and plants exposed to PAAN1135 and PAAN1181 at equal intervals of 4 days up to 16 dpi. *C* control plants, *CJAN1179* fungus with positive effect on plants, *PAAN1135* fungus with no effect on plants, *PAAN1181* fungus with negative effect on plants, *dpi* day post-inoculation

Impact of all the 150 fungal isolates on the growth and development of *A. thaliana* Col-0 was investigated using an in vitro fungus–plant interaction assay. Among all tested isolates, CJAN1179, a root endophyte from *C. jwarancusa*, influenced plant growth most positively (Fig. 1). In the presence of CJAN1179, the number of *A. thaliana* lateral roots increased significantly and a little increase in root hairs and shoot weight was noticed. For all other plant growth parameters, CJAN1179 caused negligible or no effects. On the opposite, isolate PAAN1181 was the most detrimental for the plant and isolate PAAN1135 appeared to show no effect in this regard (Fig. 1, Suppl. Fig. 1). In the subsequent detailed characterisation of CJAN1179 with respect to plant growth-promoting effects and possible phytohormone production, PAAN1181 and PAAN1135 were included in the experiments for comparison.

### The living fungus, not the culture filtrate mediates plant growth promotion

To figure out whether the fungal impact on plant development is mediated by fungal metabolites of the culture filtrate or requires the interaction of the actively growing fungus with the plant roots, effects of the three fungi on plant growth were compared between two types of inoculations FPI (living fungus) and FCFI (culture filtrate). The two treatments were found to have obvious differences (Suppl. Fig. 2).

**Fig. 2:**
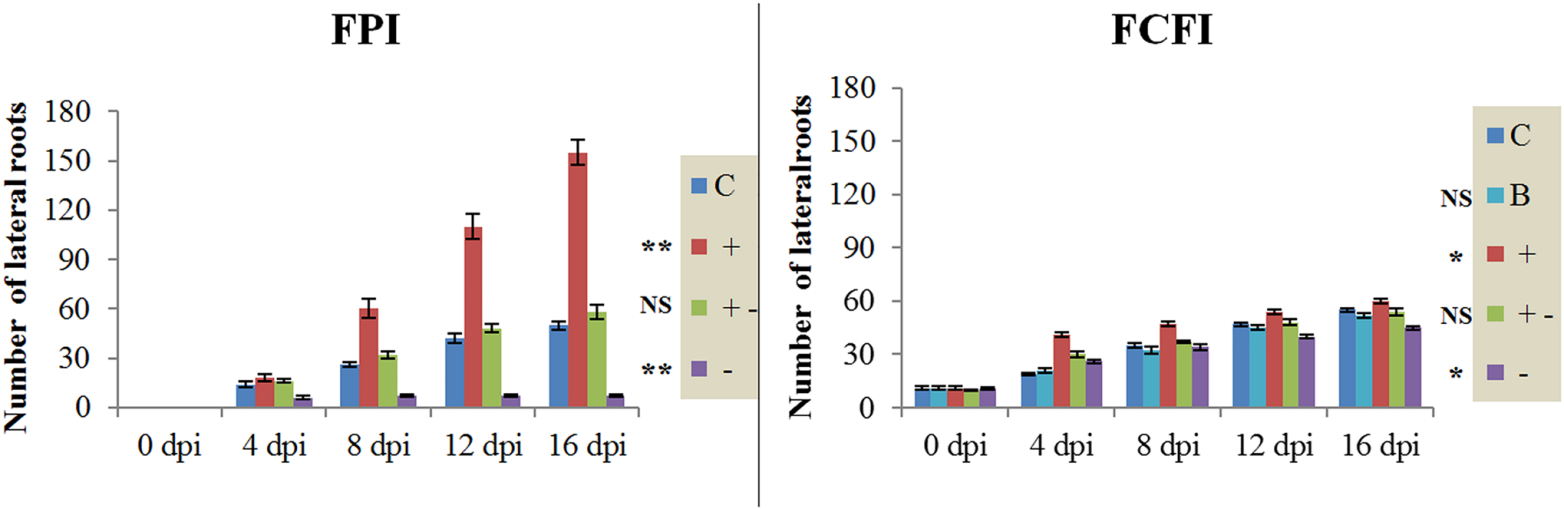
The most remarkable plant growth in the form of enhanced lateral root growth in case of direct CJAN1179-*A. thaliana* root interaction through FPI as compared to that of FCFI effect on lateral roots: FPI = fungal plug inoculation, *FCFI* fungal culture filtrate inoculation, *dpi* days post-inoculation, *C* control plants (plants without any fungal exposure), +  = CJAN1179 (fungus with positive effect on plants), + − = PAAN1135 (fungus with no effect on plants), − = PAAN1181 (fungus with negative effect on plants). The mean differences between inoculated plants and control plants; very significant (P**) at *P* ≤ 0.01, significant (P*) at *P* ≤ 0.05 and non-significant (P^NS^) at *P* > 0.05 (shown with the legend). Mean ± SE of three independent experiments, n = 30. A complete comparison of effects of CJAN1179 on plant root, shoot and flower growth between FPI and FCFI is given in Suppl. Fig. 2

CJAN1179 plug inoculation promoted plant growth most remarkably with regard to the number of lateral roots (210% increase), and showed little effect on root hairs (qualitative parameter) and shoot weight (33.3% increase) as compared to the corresponding controls but showed little or no effect on other growth parameters. Final increase in the number of lateral roots in plants exposed to CJAN1179 CF (9.1%) as compared to that of the controls was negligible when compared to that in the case of CJAN1179 plug inoculation (Fig. 2).

PAAN1181 plug inoculation caused highly negative effects on all root, shoot and flower growth (no flowers appeared in this case) parameters. In the same way, PAAN1181 CF application showed very less drastic effects on plant growth as compared to that in case of plug inoculation. PAAN1135 plug as well as CF inoculations did not mediate any change in almost all plant growth parameters with the exception that the plant leaves were yellowish at 16 dpi after PAAN1135 plug inoculation (Suppl. Fig. 2).

These observations reveal that when the model plant roots come in contact with the fungus CJAN1179, the influence on plant growth is many times greater than when the culture filtrate of the same fungus is used. An obvious increase in the number of lateral roots was observed from 0 to 16 dpi with the culture plug (FPI), however, when the culture filtrate (CF) was applied, a change in lateral root number was noticed only at 4 dpi, which thereafter became practically steady until 16 dpi.

### CJAN1179 and PAAN1181 showed statistically significant effects on plant growth

Effects of the three fungi on *A. thaliana* growth in reference to quantitative parameters were statistically analysed. CJAN1179 plug inoculation showed very significant P** (*P* < 0.01) positive effects on the number of lateral roots, number of leaves, rosette diameter and shoot weight in these parameters but did not show any effect *P*^NS^ (*P* > 0.05) on other growth parameters, e.g. length of primary root (*P* = 0.133) and flower stalk length (*P* = 0.149). CJAN1179 CF showed significant positive effect P* (*P* < 0.05) only on number of lateral roots (*P* = 0.020) and number of leaves (*P* = 0.021) and did not show any effect *P*^NS^ (*P* > 0.05) on other growth parameters. In contrast, PAAN1181 plug inoculation caused very significant P** (*P* < 0.01) negative effects on all root, shoot and flower growth parameters. PAAN1181 CF caused significant negative effects P* (*P* < 0.05) on plant growth whereas PAAN1135 plug as well as CF inoculation did not show any significant change *P*^NS^ (*P* > 0.05) in plant growth.

In statistical analysis, mean differences between inoculated and control plants for various plant growth metrics indicate the extent or intensity of fungal effect on plant growth, which can be seen as differences between the lines and bars of graphs. Thus, according to these mean differences, CJAN1179 had a significant favourable effect only on number of lateral roots when active fungal plug inoculation was used (Fig. 2).

### CJAN1179 produces IAA

The most remarkable beneficial effect of CJAN1179 on plant growth was found to be a significant increase in the number of lateral roots. The phytohormone auxin, most predominantly occurring as IAA, is an important player in the formation of lateral roots (Idris et al. 2007). Thus, we hypothesised that CJAN1179 is generating its plant growth promotion effect through production and secretion of IAA.

The presence of IAA in the CF of the three fungi was detected using Salkowski’s test. During this test, pure IAA results in a pink colour but red colour indicates the production of tryptophol (TOL) along with IAA from tryptophan (Robinson et al. 1998; Rahman et al. 2010). TOL has been reported as a by-product formed during the conversion of L-tryptophan to IAA through the indole-3-pyruvate pathway of tryptophan-dependent production of IAA and is convertible to IAA (Furukawa et al. 1996; Maor et al. 2004). The reaction mixture containing CJAN1179 CF grown in the absence of tryptophan showed no colour change indicating that there is no or very low IAA production in the absence of tryptophan. In case of CJAN1179 cultured in the presence of tryptophan, the CF changed colour of the Salkowski’s reagent to red. This shows that CJAN1179 produces IAA and possibly TOL when grown in the presence of tryptophan.

Evaluation using colourimetric assay at 530 nm revealed that in the absence of tryptophan, CJAN1179 only was able to generate extremely modest levels of IAA. That is why no significant colour change was visible.

However, CJAN1179 was able to produce a lot of IAA (1638 µg/ ml) in the presence of tryptophan.

**Fig. 3:**
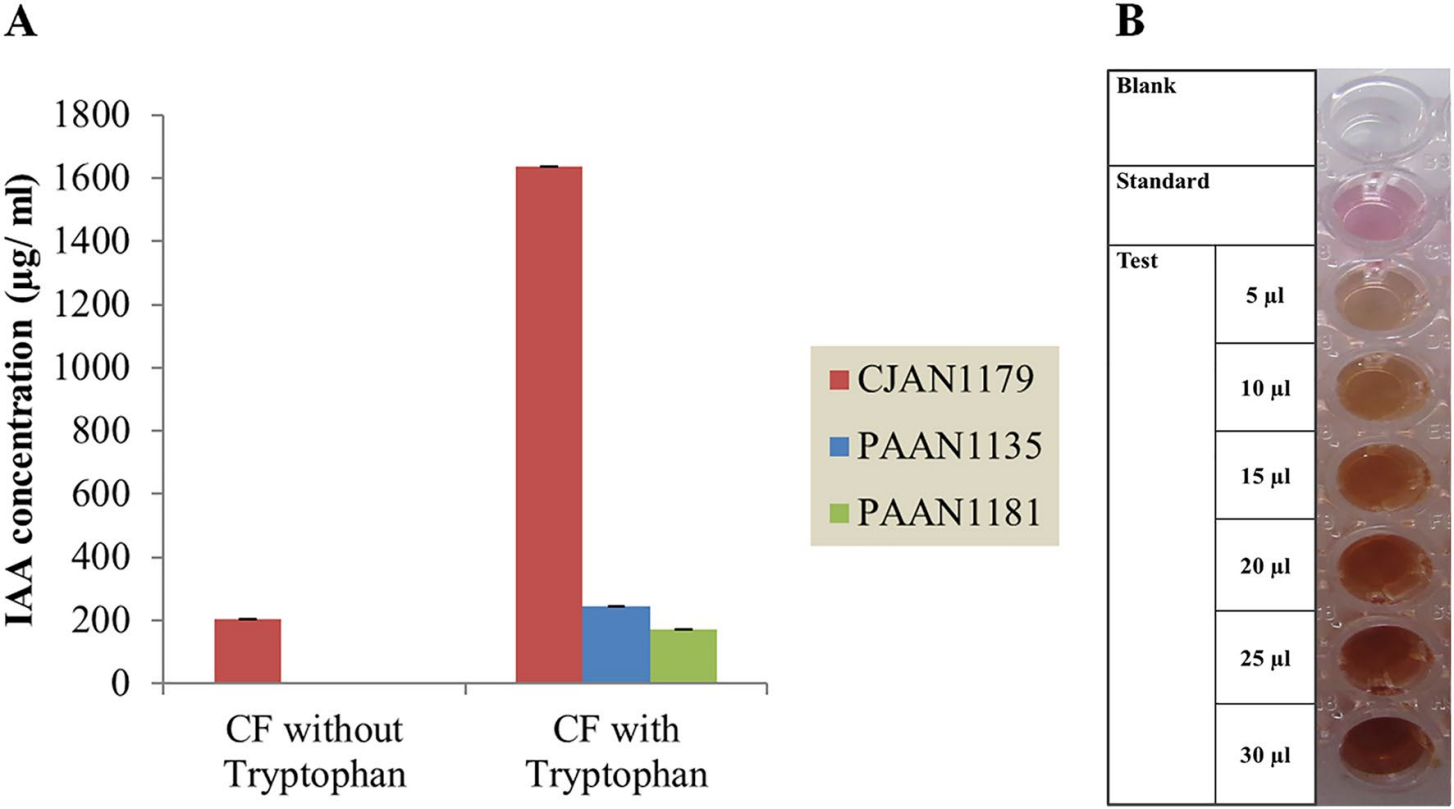
**A** IAA concentration of the culture supernatant of the three fungi grown with and without tryptophan. *CF* fungal culture filtrate. Using OD values of fungal culture filtrates, concentration of auxin was calculated through standard curve formula X = Y + 0.010/ 0.018 (X = conc. of IAA to be calculated, Y = mean ± SE of three replicates of OD values for each assay in triplicate, *n* = 9), **B** colour change in reaction mixture having CJAN1179 CF with tryptophan after incubation, Blank = reaction mixture without standard or test sample, Standard = 0.01% Indole-3-acetic acid solution, Test = reaction mixture having different volumes (5–30 µl) of sample (CJAN1179 CF with 0.1% L-tryptophan). Reaction mixture = Test/ standard/blank + H_2_O + Salkowski reagent = 250 µl

Under similar conditions, PAAN1135 and PAAN1181 produced very low IAA amounts of 304 and 172 µg/ ml, respectively, and that only in the presence of tryptophan (Fig. 3).

### CJAN1179 belongs to the *Sordariomycetidae*

#### Molecular characterisation and phylogenetic analysis of CJAN1179

**Fig. 4:**
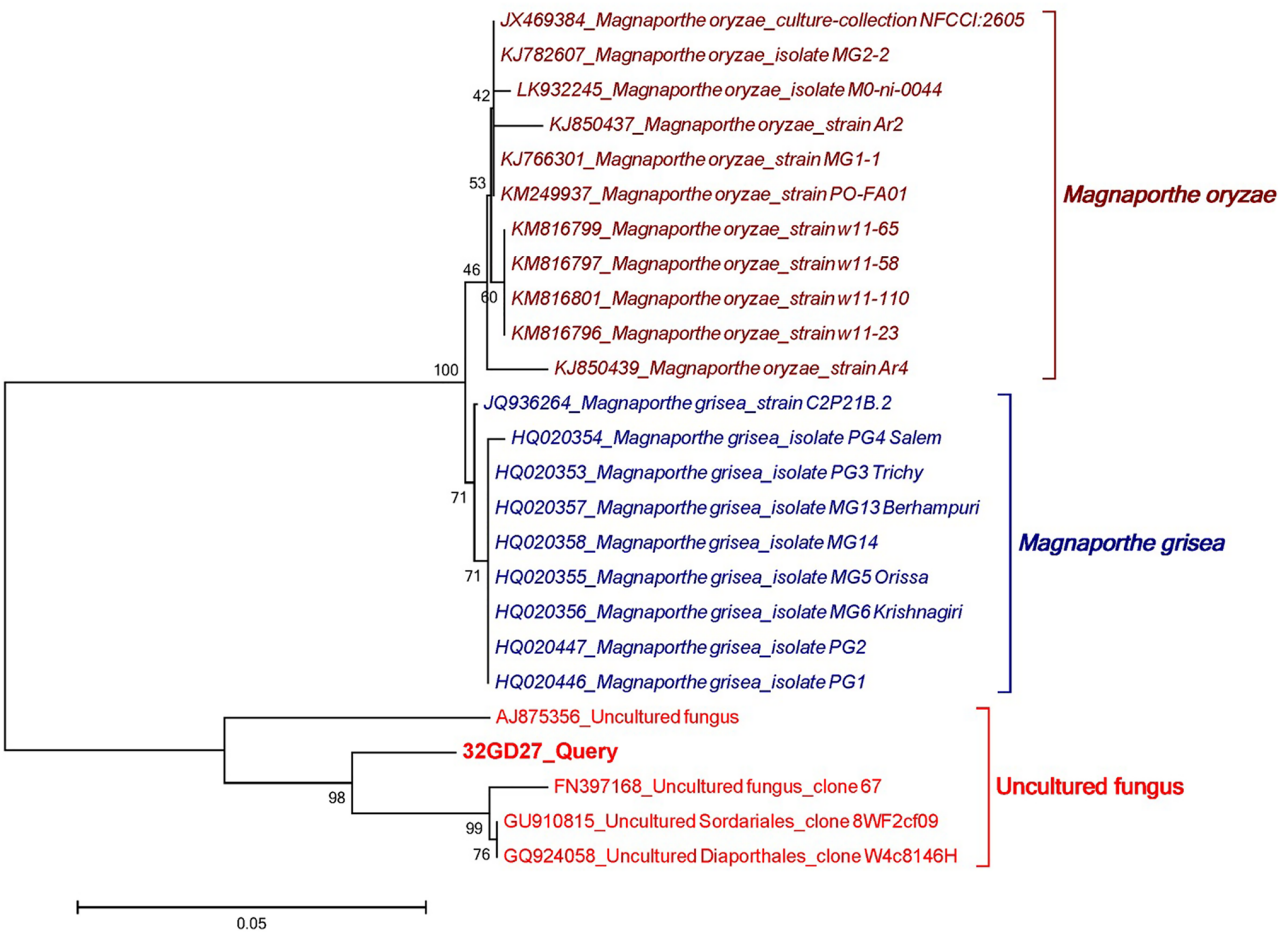
Phylogenetic comparison of CJAN1179 (32GD27_Query) ITS sequence with corresponding sequences of selected fungal species: The query sequence is clearly separated from the clades of *Magnaporthe oryzae* and *Magnaporthe grisea* and fall into the clade of four uncultured fungi, two of them are unclassified and other two are uncultured *Sordariales* and uncultured *Diaporthales*

The obtained ITS sequence of CJAN1179 is available at NCBI, accession number LC198062. The phylogenetic tree is representing 3 different clades (monophyletic groups), i.e. *Magnaporthe oryzae*, *Magnaporthe grisea* and uncultured fungi (Fig. 4). Bootstrap values are supporting the analysis. Using BLAST, the query sequence was found to match best with the corresponding sequences of four uncultured fungi with the GenBank accession nos. **AJ875356**, **FN397168**, **GU910815** (Sordariales) and **GQ924058** (Diaporthales) (Suppl. Fig. 3). Query coverage is 100% with 1% gaps and sequence identity between 92 and 94%.

### Morphological characterisation

**Fig. 5:**
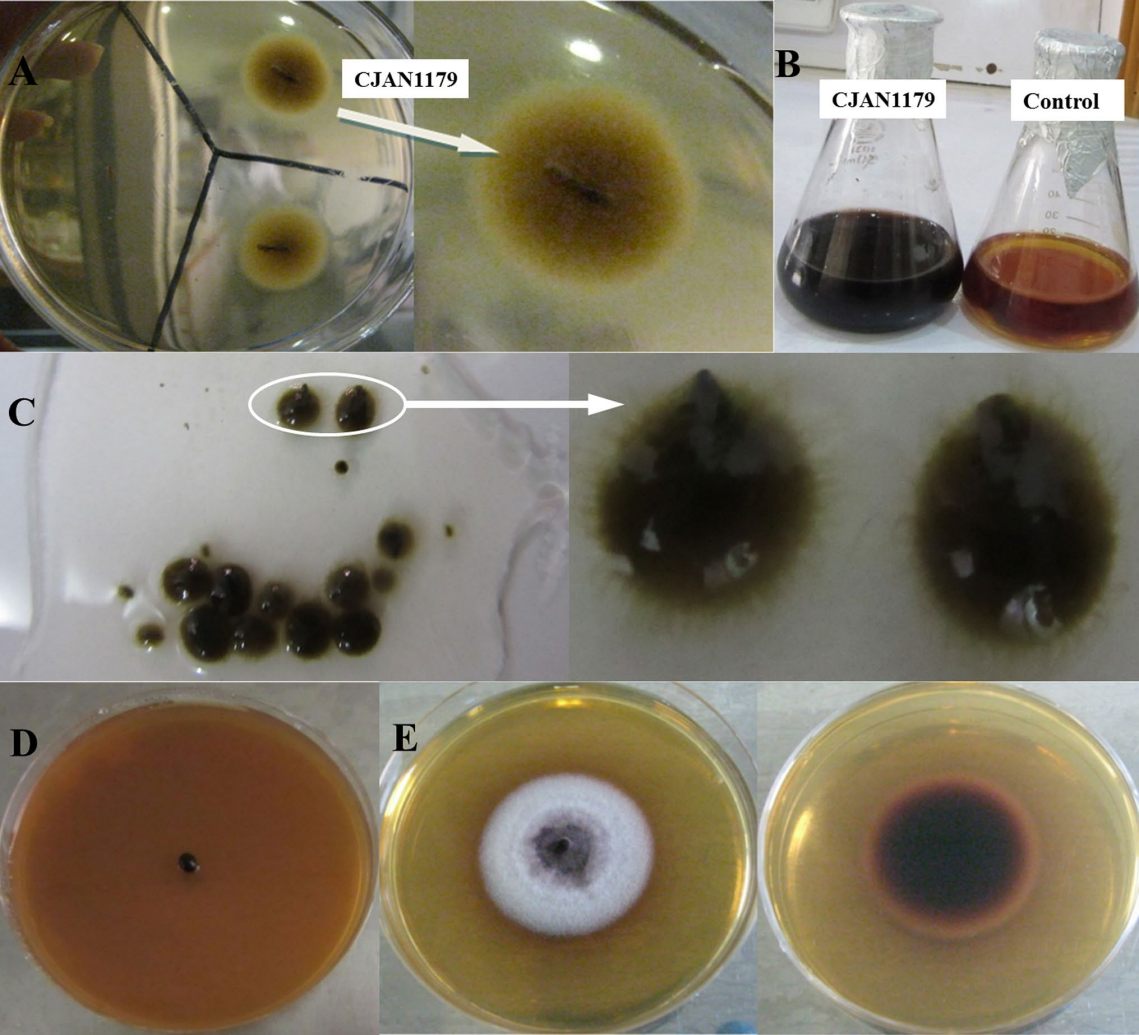
**A** Shape of fungus CJAN1179 at the time of isolation from a root piece of *C. jwarancusa*, **B** cultivation of isolated fungal mycelial colony in SB causes the broth to acquire a deep cherry red colour. **C** In liquid culture, fungal mycelia turn into smooth and hairy black round sporocarps (fruiting bodies). Fully mature sporocarps are flask-shaped forming beak-like structures called stromata (highlighted on glass slide and shown in zoom). **D** A single stroma can be cultivated to a fully grown fungal mycelial colony on the agar plate and thus completes and continues the life cycle. **E** Front and backside view of a fully grown CJAN1179 colony on the agar plate

Following a molecular phylogenetic analysis of CJAN1179’s ITS sequence for taxonomic lineage identification, a fungal colony produced on SA plate and spores or fruiting bodies developed in SB media were subjected to a macroscopic examination. CJAN1179 produced a yellowish-brown mycelial colony and turned SB to a deep cherry red coloured medium indicating the secretion of colourful metabolites. The fungus produces smooth, hairy black spherical sporocarps in SB. The fully mature sporocarps appear to be flask-shaped with a beak-like structure. These sporocarps can generate a fully grown fungal colony on SA medium. A sporocarp first transforms into a little yellowish-brown fungal mycelia colony, which then matures into a purplish-grey colony, turning the agar medium cherry red (Fig. 5).

## Discussion

Because of plant–microbe interactions, the plant–soil interface is thought to be a hotbed of biological activity (Morgan et al. 2005). Plants thriving in a variety of environmental settings including deserts appear to be linked with endophytic and rhizospheric microorganisms that are thought to be involved in a number of biological processes enabling these plants to survive in harsh climates (Cherif et al. 2015; Makhalanyane et al. 2015). Below-ground microflora, which includes plant growth-promoting fungi (PGPF), is, therefore, critical for establishment of vegetation and a focus for investigators exploring sustainable development strategies. Keeping this hypothesis in mind, we have isolated rhizospheric and root-endophytic fungi from plants growing in the Cholistan desert and screened them for their plant growth-promoting potential. Using *A. thaliana* as a model plant, we investigated plant growth-promoting features of 150 fungal isolates and identified an extremely effective PGP fungus CJAN1179. When compared to unexposed plants, this fungus has a considerable impact on root formation and increased the number of lateral roots many folds.

A variety of phytohormones, such as auxin, cytokinins and gibberellins, are involved in different aspects of plant growth (El-Showk et al. 2013). They are produced by plants in response to environmental signals and are required in large quantities to maintain normal plant growth under stressful conditions (Waqas et al. 2012). Several bacteria and fungi engaged in plant–microbe interactions have been shown to produce phytohormones such as IAA to improve plant water and nutrient uptake by altering root architecture (Hamayun et al. 2010). *Trichoderma* species are an example such fungi that have been studied extensively for their potential function in plant growth and survival (Bais et al. 2004; Hermosa et al. 2012). Auxin is known to be responsible for the start and production of lateral roots formed in the post-embryonic stage by several cell divisions because of cell cycle activation in “founder cells” present in the pericycle region, while cytokinins and gibberellins are mostly responsible for shoot growth (Glick 2012). Since environmental stimulations increase auxin production and so enhance lateral root formation, auxin plays a direct role in lateral root formation (Casimiro et al. 2001; Ortíz-Castro et al. 2009). Through auxin and ethylene-related activities, root hair production is also linked to the enhancement of plant root architecture for improved water and nutrient uptake from the soil (Masucci and Schiefelbein 1994; Grierson et al. 2014). We hypothesised that CJAN1179 may be exerting its plant growth-promoting effect, directly or indirectly, through one of these phytohormone. Since CJAN1179 leads to an increase of lateral roots, particularly in case of FPI, we assumed that exposure of *A. thaliana* to CJAN1179 either generates the necessary trigger to escalate production of the required phytohormone by the plant or alternatively, CJAN1179 may produce large amounts of the required phytohormone by itself and provides it to the associated plant as a result of fungal–plant interaction. Such comparative effects on the number of lateral roots affected by FPIs and FCFIs have been reported in the literature (Felten et al. 2010).

The naturally occurring prominent auxin, IAA is generated via multiple mechanisms in plants and microorganisms (Gruen 1959; Spaepen and Vanderleyden 2011). The mechanism of auxin production is known only for very few fungi. The major pathway for IAA production in fungi is tryptophan-dependent where tryptophan released from the plant is used by fungi as the main precursor for the production of IAA.

**Fig. 6:**
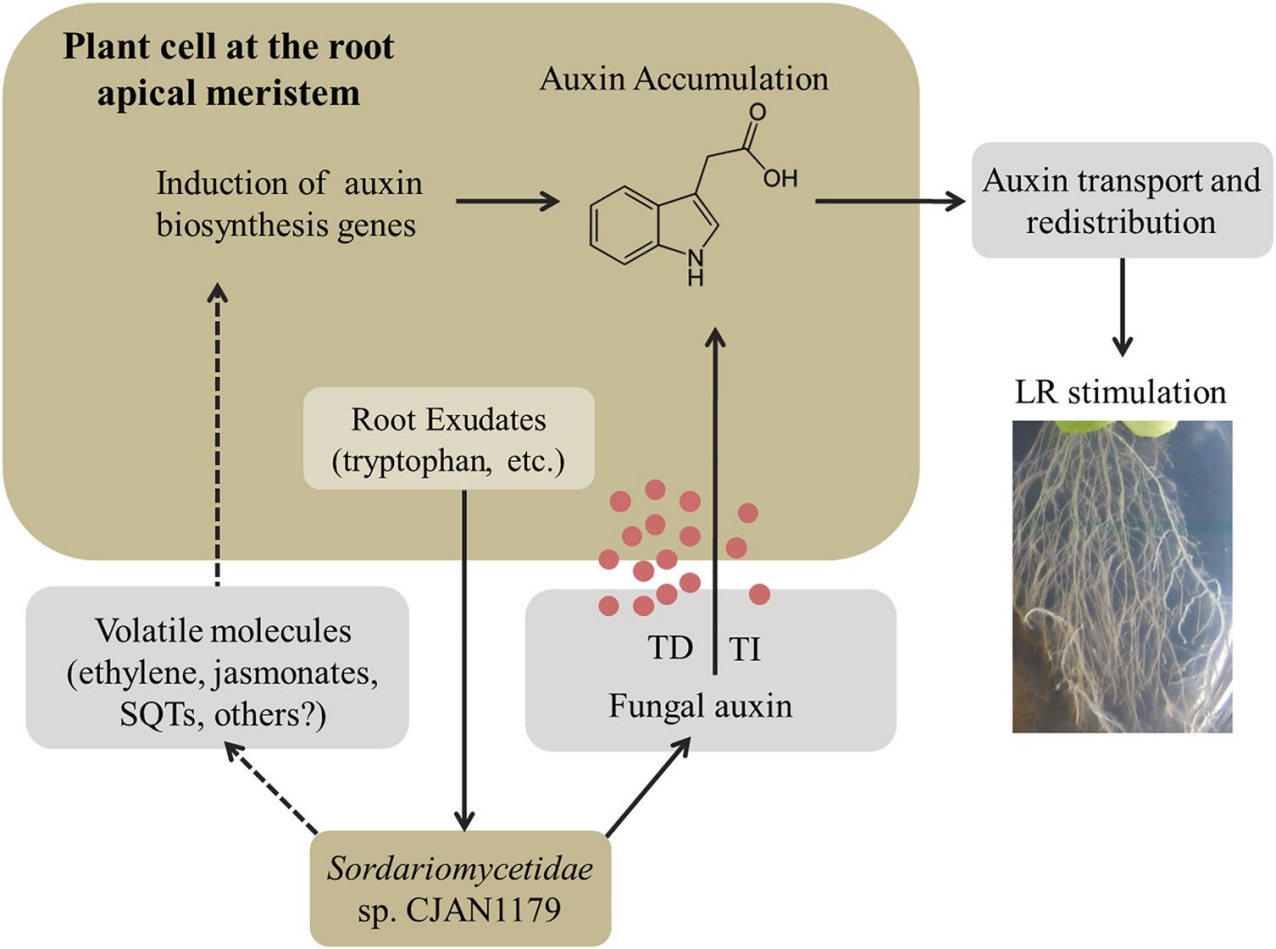
CJAN1179 auxin production via a tryptophan-depended (TD) mechanism is much higher than by tryptophan-independent (TI) mechanism concluded by current work and displayed in straight line. As described for other fungi, it might be that auxin production by the plant is stimulated by fungal volatiles. This putative fungus–plant interaction signalling is displayed in dotted lines and has been proposed for future work. CJAN1179 might produce certain volatiles such as ethylene, jasmonate and sesquiterpenes (SQTs) that activate plant auxin biosynthesis genes. Both auxin production mechanisms are likely involved in enhancing lateral root and root hair formation in *A. thaliana*. Model adopted and modified from Felten et al. (2010)

Tryptophan-independent production of IAA has also been reported where indole-3-acetamide (IAM) is converted into IAA (Furukawa et al. 1996; Robinson et al. 1998; Larekeng et al. 2019). Endophytic fungi, pathogen or PGP, are known to produce higher amounts of IAA than free-living fungi (Fouda et al. 2015). IAA has been suggested to serve as a communication signal between the endophytic fungi and the host plant roots (Mehmood et al. 2018; Mehmood et al. 2019) and it is known to affect gene expression in the target organism (Fu et al. 2015). Thus, being an endophyte, we expected CJAN1179 to produce IAA. Our results show that CJAN1179 indeed produces IAA along with some amount of TOL as a by-product of the tryptophan-dependent pathway. This pathway is known to involve indole-3-pyruvic acid (Furukawa et al. 1996). Further, production of excessive lateral roots in the plants exposed to CJAN1179 may also indicate the involvement of volatiles that activate the genes for enzymes related to auxin biosynthesis by the plant although this aspect was not further investigated (Fig. 6). Ethylene and jasmonate, for instance, have been reported as such kind of volatiles (Hermosa et al. 2012). Our results show that this fungus, CJAN1179, is a mutualistic fungus that promotes plant growth by producing IAA, which mediates lateral root growth via a tryptophan-dependent mechanism. Tryptophan, the main precursor of IAA, is secreted by plants during fungal–plant interaction (Felten et al. 2010).

We sequenced the ITS regions of nuclear rRNA for the molecular characterisation of CJAN1179 because the ITS regions in fungal genomes are highly variable among species and even among the entities of the same species of fungi (Liew et al. 1998). This region, therefore, is the source of maximum level of identification among fungal populations (Tarbell 2008; Schoch et al. 2012). CJAN1179 was subjected to molecular characterisation using multiple sequence alignment of the obtained ITS sequence (Suppl. Fig. 3) and phylogenetic analysis (Fig. 4). CJAN1179 was categorised as a member of the *Sordariomycetes*. BLAST analysis of our query sequence gave hits with four uncultured fungi in the database. Two of these uncultured fungi with accession numbers GU910815 and GQ924058 have been classified up to order levels, *Sordariales* and *Diaporthales*, respectively, while the other two are unclassified. Length of the branches of the two clades, i.e. *Magnaporthe oryzae* and *Magnaporthe grisea*, under the order *Magnaporthales*, clearly separate our query sequence from these and lead to it being categorised in the clade of the uncultured fungi. Based on the maximum sequence similarity with the uncultured *Sordariales* and *Diaporthales* isolates we have classified our cultured fungus up to the subclass level, i.e. *Sordariomycetidae* of class *Sordariomycetes*, where it possibly represents a new species under the order *Sordariales* or *Diaporthales*. The *Sordariomycetes* are characterised by the production of unitunicate (single-walled), inoperculated (without operculum or lid) asci inside perithecal (flask-shaped) fruiting bodies. *Sordariomycetes* from different habitats are reported to live as pathogens and endophytes of plants, mammals and arthropods (Zhang et al. 2006; Maharachchikumbura et al. 2015). Our query sequence suggested that CJAN1179 either belongs to the *Sordariales* or the *Diaporthales*. Based on background knowledge about *Sordariomycetidae* (Maharachchikumbura et al. 2016) and macroscopic studies with CJAN1179 showing flask-shaped black sporocarps with beak-like structure, we suggest CJAN1179 to be a member of the order *Diaporthales* where CJAN1179 appears to be a new species. Thus, according to the phylogenetic analysis following taxonomic lineage can be drawn for CJAN1179: Eukaryota > Fungi > Dikarya > Ascomycota > Pezizomycotina > Sordariomycetes > Sordariomycetidae > Unclassified Sordariomycetidae (probably Diaporthales). The fungus has thus been named as *Sordariomycetidae* sp. CJAN1179 and the obtained ITS sequence is publicly available (GenBank, accession no. **LC198062**). On the basis of sequence analysis of CJAN1179 ITS region, we have classified the isolate as a new member of the *Sordariomycetidae*.

## Conclusion

We demonstrate the role of a root-endophytic fungus CJAN1179 during fungus–plant interaction. The fungus mediates remarkable growth promotion of *A. thaliana (Col-0)* through enhanced lateral root growth. It is capable of producing high amounts of IAA in the presence of tryptophan utilising the tryptophan-dependent indole-3-pyruvate pathway. We believe that during direct fungus–plant interaction, CJAN1179 uses tryptophan secreted by the plant to synthesise IAA which stimulates lateral root formation. The above-mentioned interaction between CJAN1179 and *A. thaliana* is depicted in a simple hypothetical model (Fig. 6), which was inspired from Felten and co-workers, who studied the beneficial fungus, *Laccaria bicolour* (Felten et al. 2010).

More research is needed to understand the molecular and physiological elements of the CJAN1179–plant interaction, as well as to look into the fungus’ ability to boost plant development in the field. It will also be important to investigate the role this fungus may play in the management of biotic and abiotic stresses in commercially important crops. Information gleaned from such investigations can aid to explore the agricultural potential of arid and wastelands when utilising fungi like CJAN1179.

## Supplementary Information

Below is the link to the electronic supplementary material.

Supplementary file1 (TIF 567 KB)

Supplementary file2 (TIF 547 KB)

Supplementary file3 (TIF 385 KB)

Supplementary file4 (TIF 684 KB)

Supplementary file5 (TIF 697 KB)

Supplementary file6 (TIF 413 KB)

Supplementary file7 (TIF 990 KB)

Supplementary file8 (TIF 936 KB)

Supplementary file9 (TIF 1001 KB)

Supplementary file10 (TIF 571 KB)

Supplementary file11 (TIF 452 KB)

Supplementary file12 (TIF 308 KB)

Supplementary file13 (TIF 577 KB)

Supplementary file14 (TIF 600 KB)

Supplementary file15 (TIF 401 KB)

Supplementary file16 (TIF 675 KB)

Supplementary file17 (TIF 507 KB)

Supplementary file18 (TIF 447 KB)

Supplementary file19 (TIF 7814 KB)

## Data Availability

Supplementary data are being submitted with the manuscript. Further material will be made available if and when required.
