# Supplementary figures and images for: A new endophytic fungus CJAN1179 isolated from the Cholistan desert promotes lateral root growth in Arabidopsis and produces IAA through tryptophan-dependent pathway
Source: Arch Microbiol. 2022 Feb 17;204(3):181. doi: 10.1007/s00203-022-02768-2 (PMC8854254; doi:10.1007/s00203-022-02768-2)

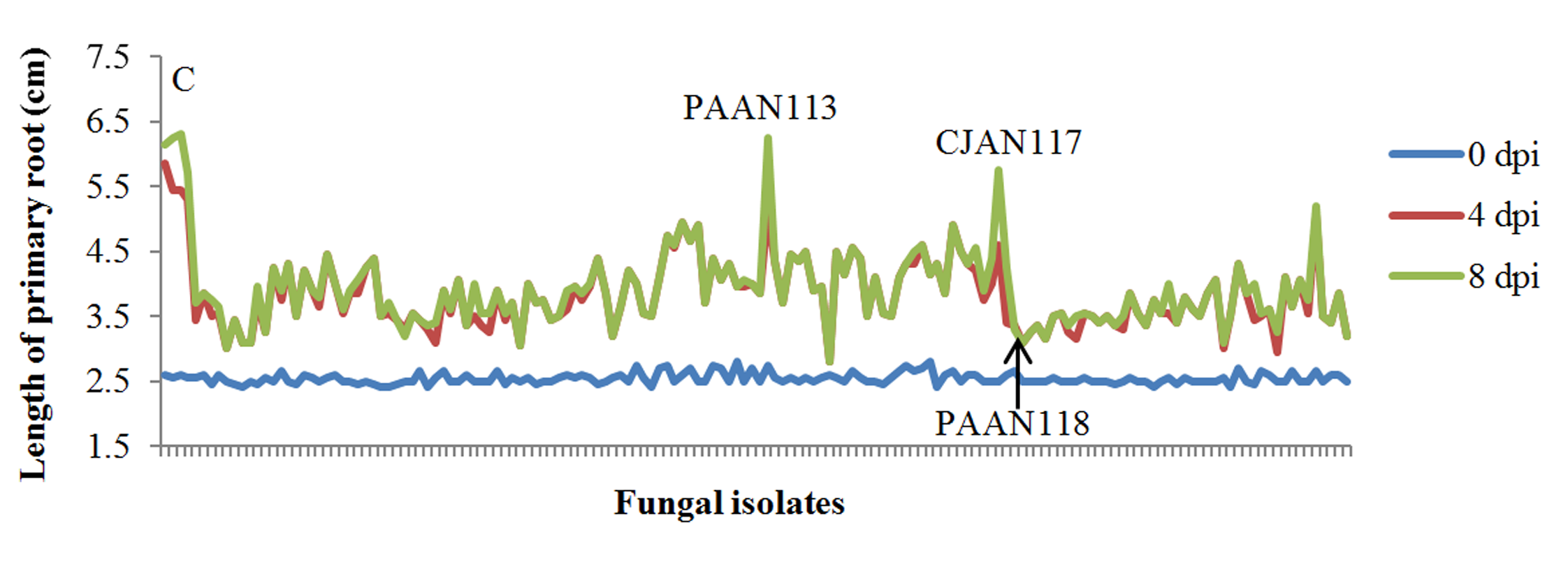

Supplement: Supplementary file 1 — Supplementary file1 (TIF 567 KB) [file 203_2022_2768_MOESM1_ESM.tif]

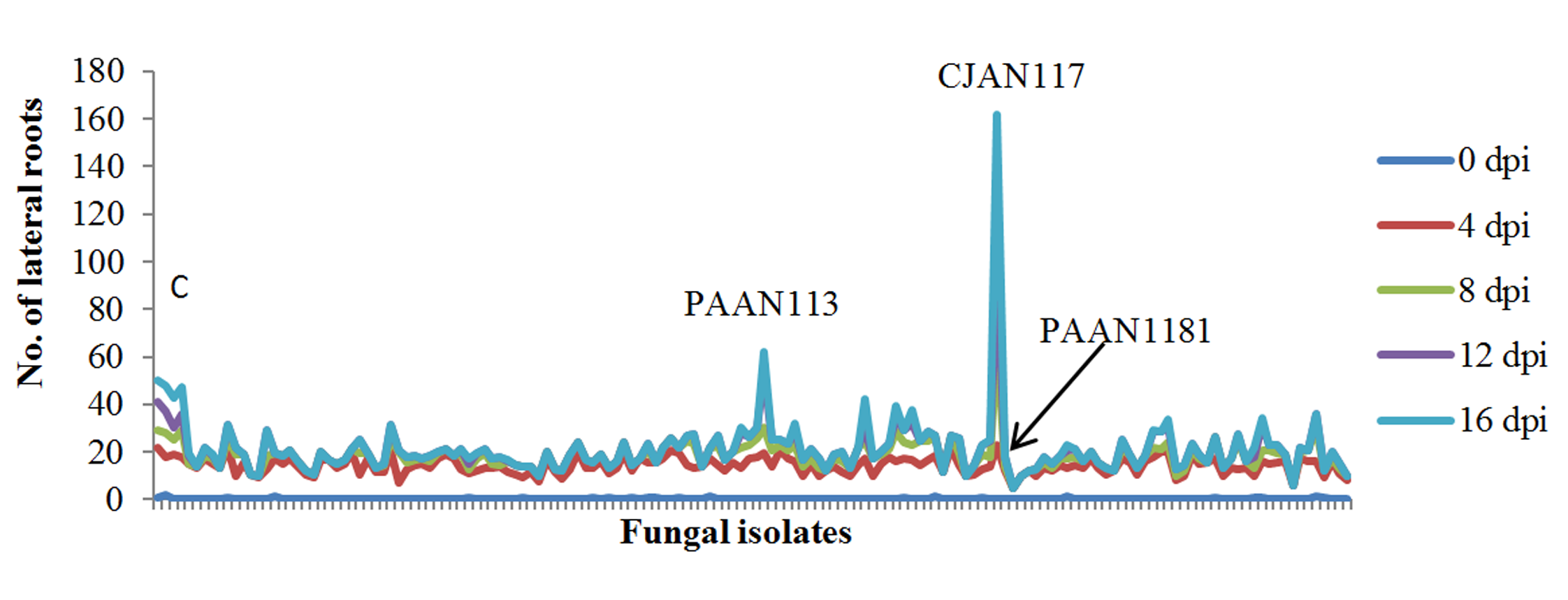

Supplement: Supplementary file 2 — Supplementary file2 (TIF 547 KB) [file 203_2022_2768_MOESM2_ESM.tif]

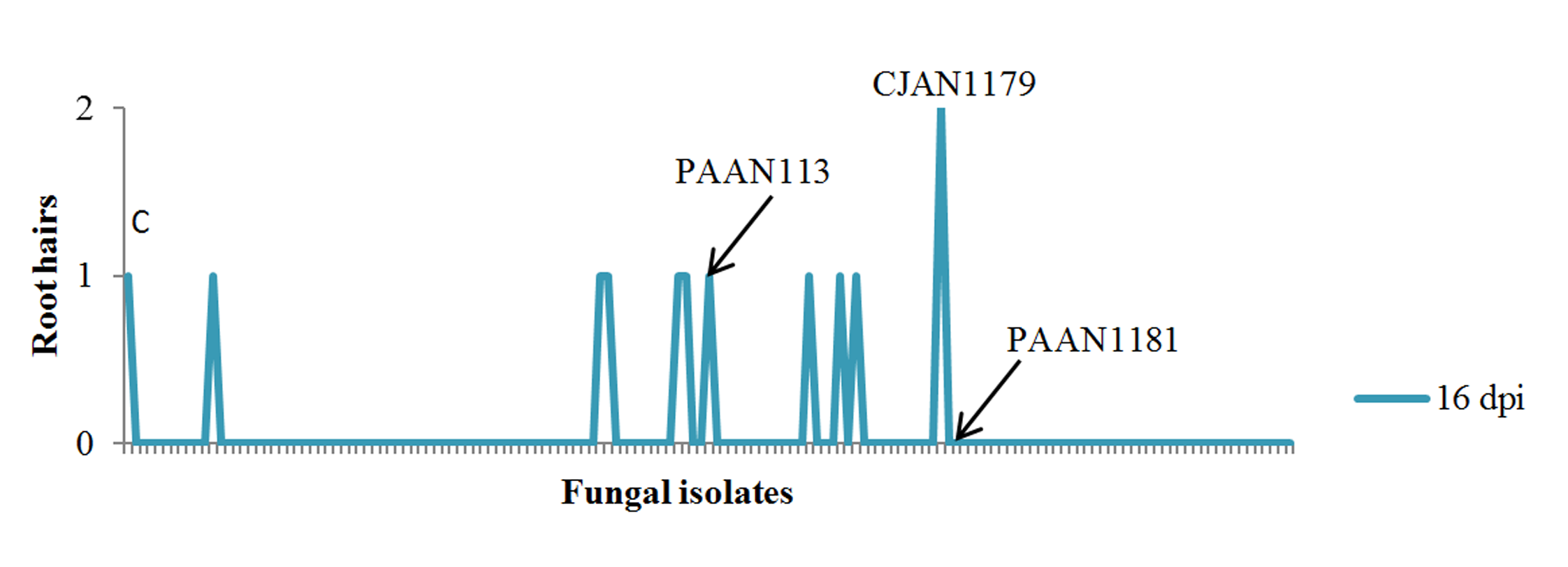

Supplement: Supplementary file 3 — Supplementary file3 (TIF 385 KB) [file 203_2022_2768_MOESM3_ESM.tif]

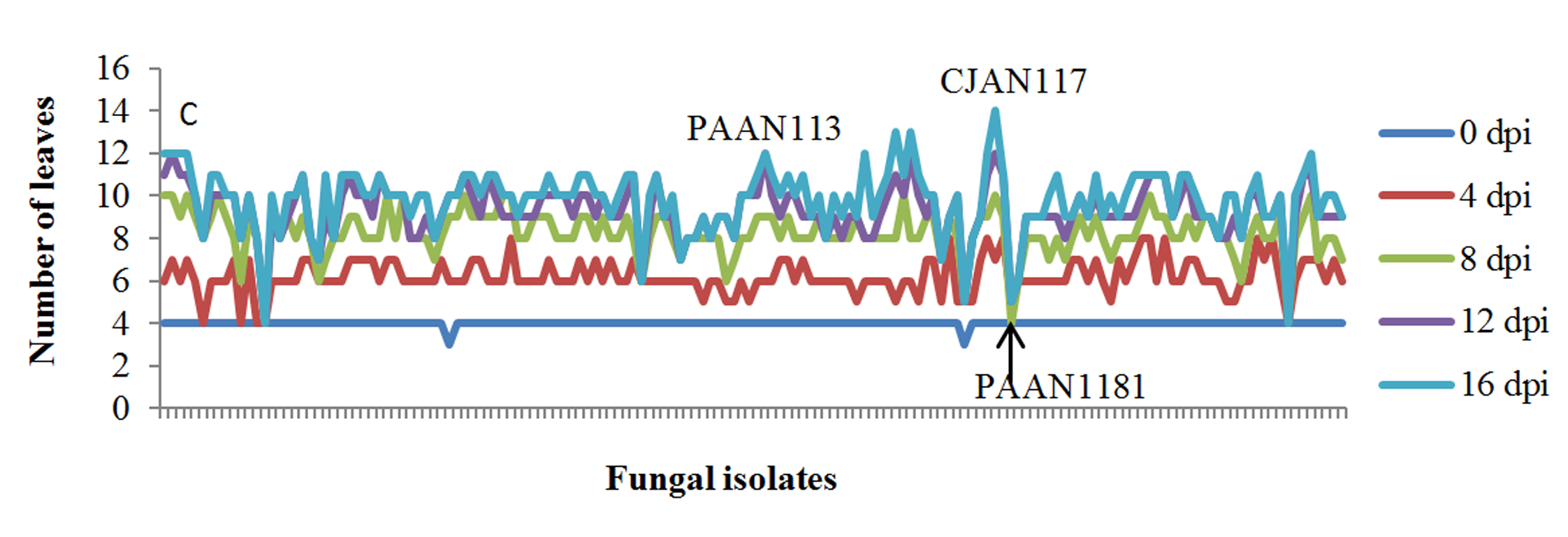

Supplement: Supplementary file 4 — Supplementary file4 (TIF 684 KB) [file 203_2022_2768_MOESM4_ESM.tif]

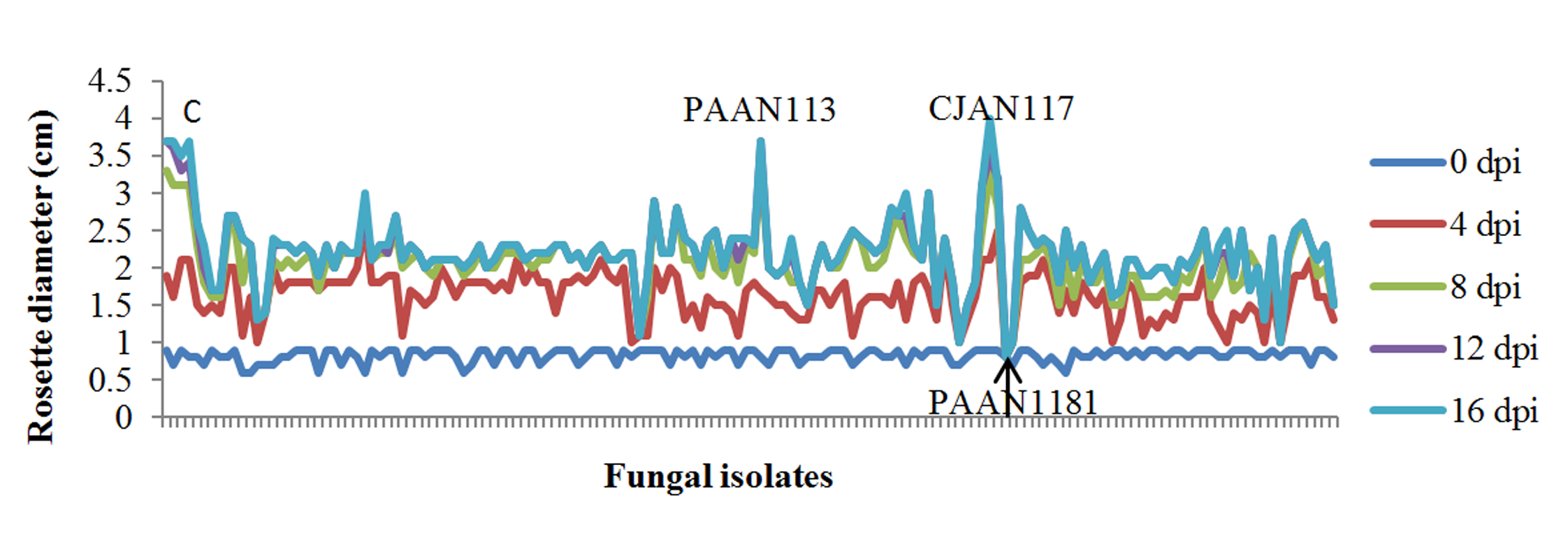

Supplement: Supplementary file 5 — Supplementary file5 (TIF 697 KB) [file 203_2022_2768_MOESM5_ESM.tif]

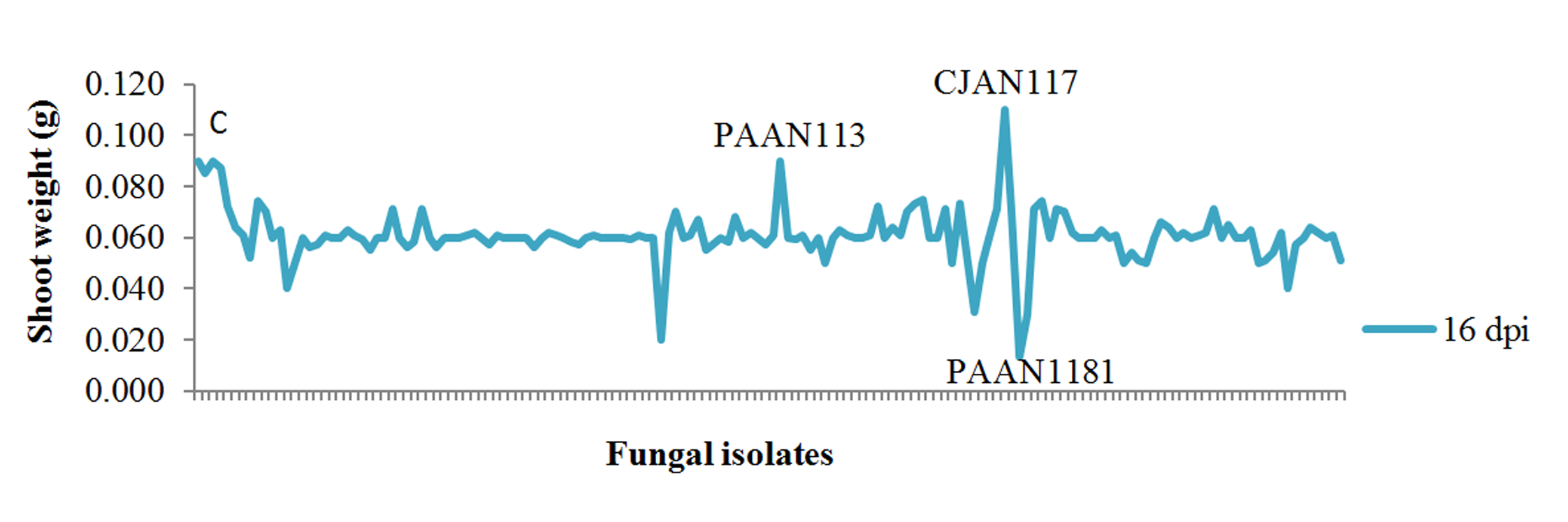

Supplement: Supplementary file 6 — Supplementary file6 (TIF 413 KB) [file 203_2022_2768_MOESM6_ESM.tif]

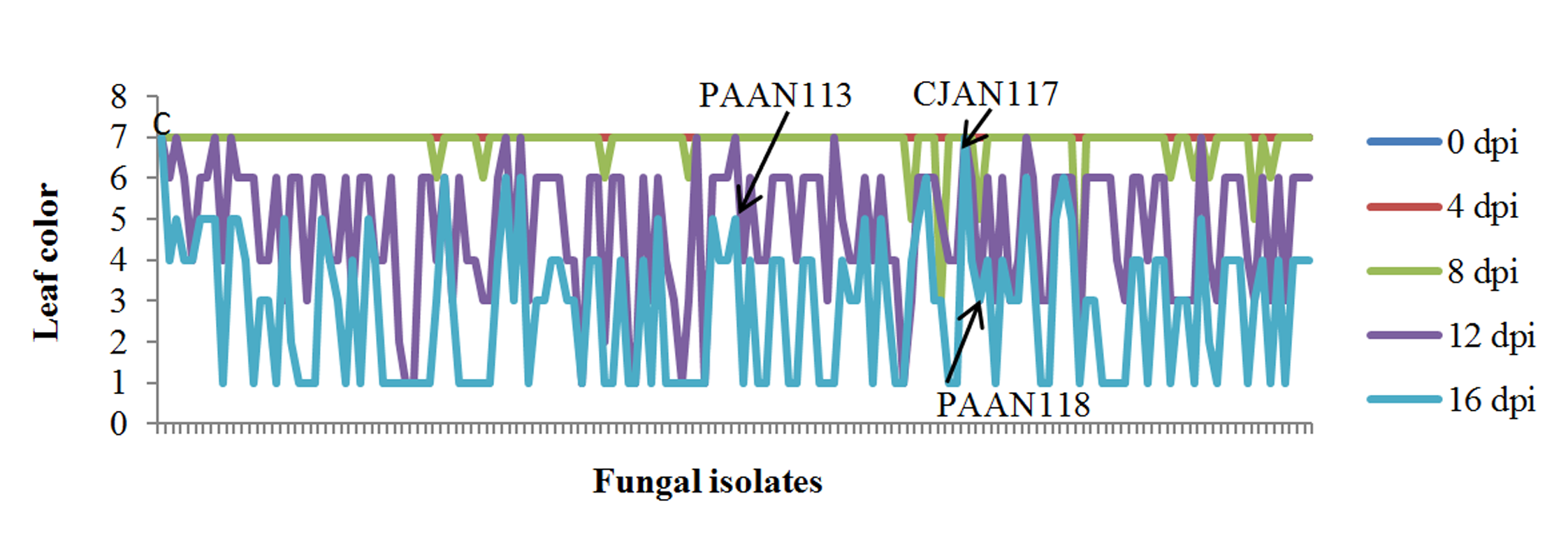

Supplement: Supplementary file 7 — Supplementary file7 (TIF 990 KB) [file 203_2022_2768_MOESM7_ESM.tif]

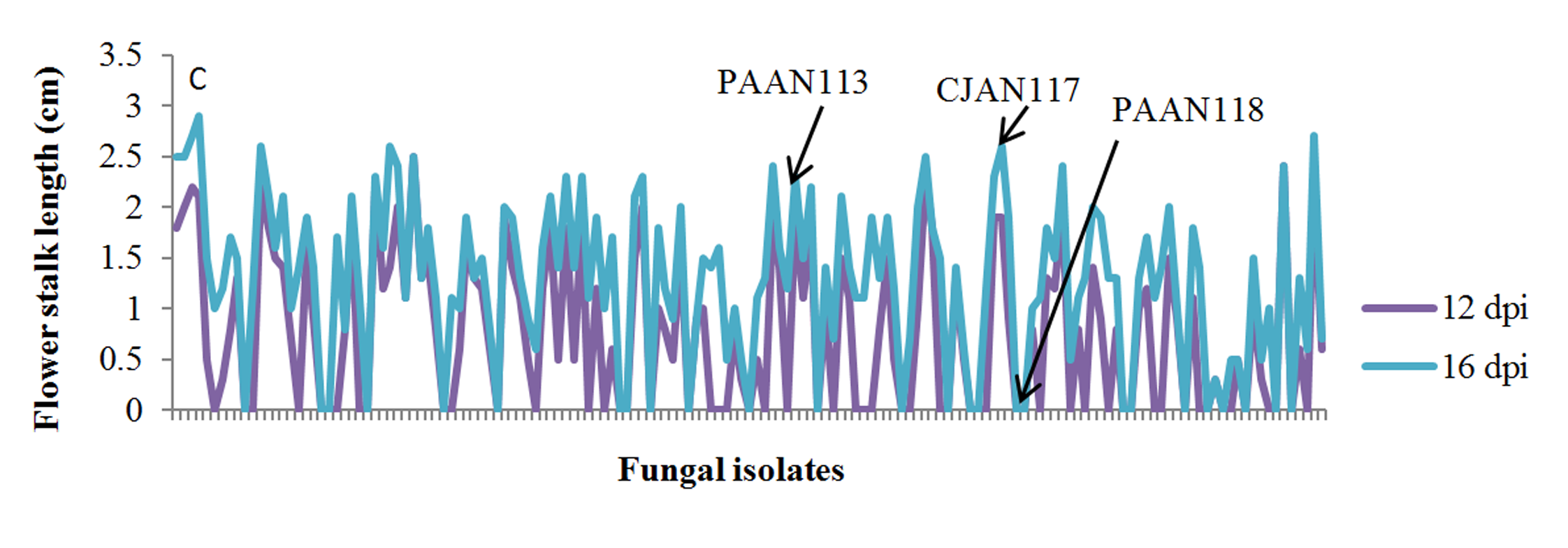

Supplement: Supplementary file 8 — Supplementary file8 (TIF 936 KB) [file 203_2022_2768_MOESM8_ESM.tif]

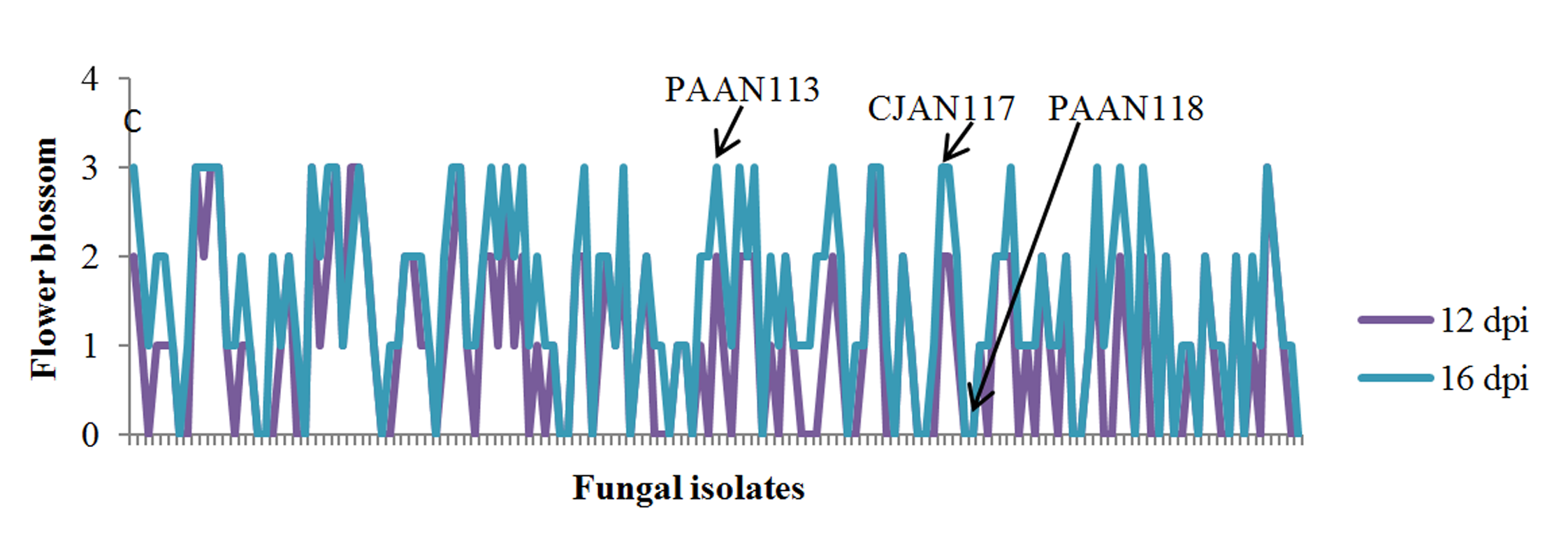

Supplement: Supplementary file 9 — Supplementary file9 (TIF 1001 KB) [file 203_2022_2768_MOESM9_ESM.tif]

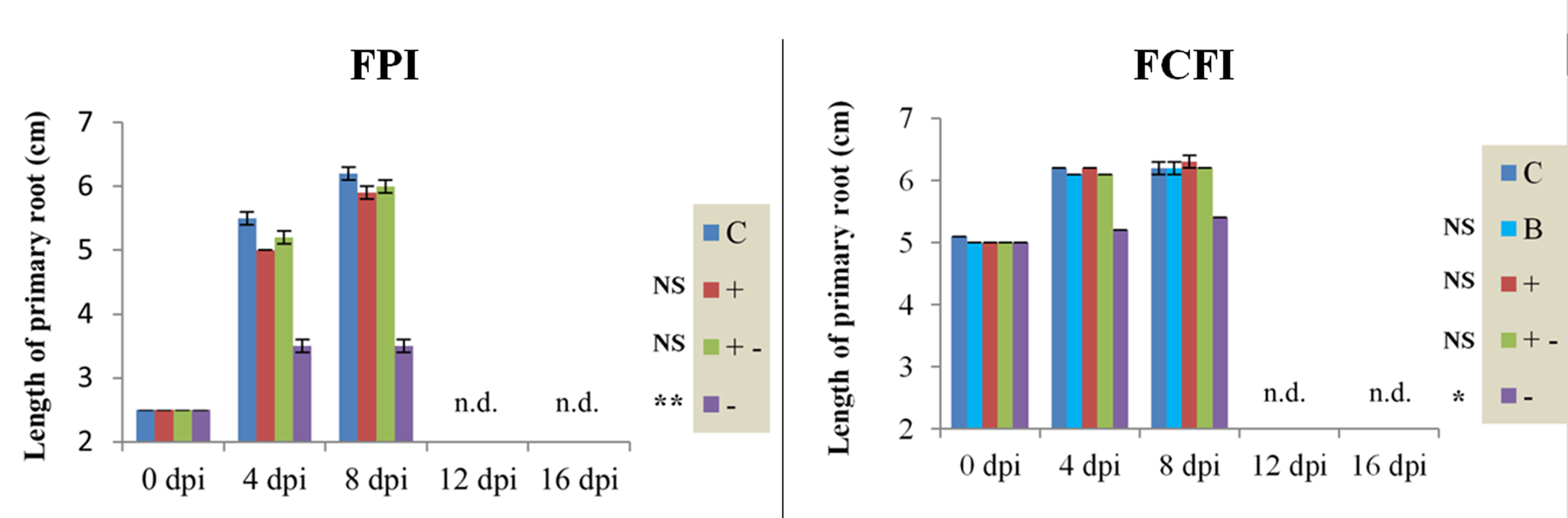

Supplement: Supplementary file 10 — Supplementary file10 (TIF 571 KB) [file 203_2022_2768_MOESM10_ESM.tif]

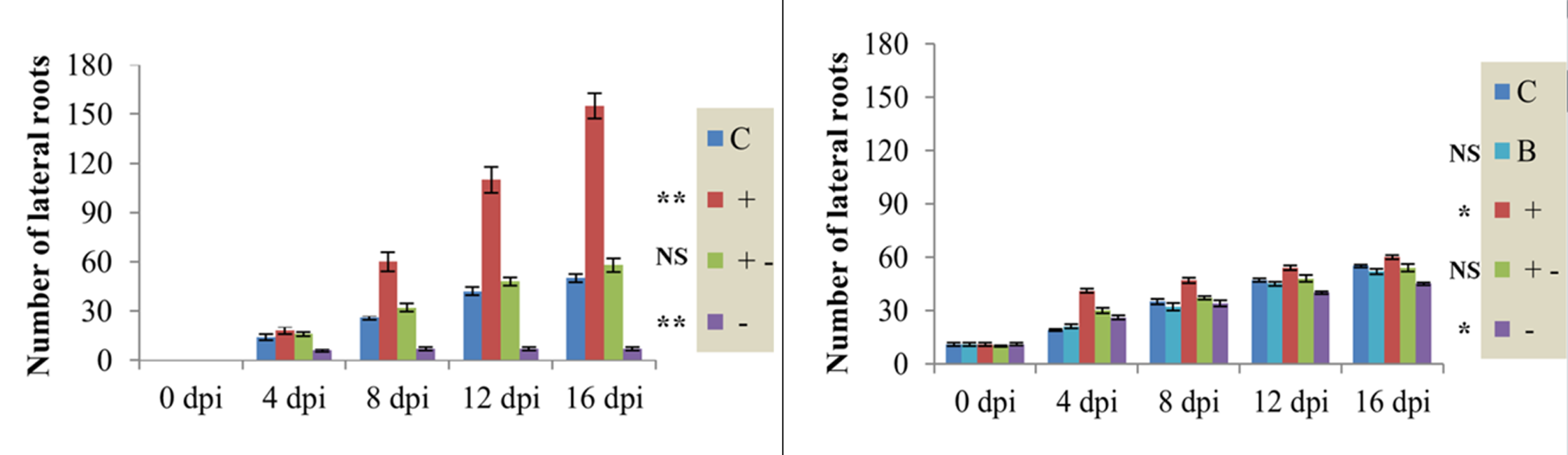

Supplement: Supplementary file 11 — Supplementary file11 (TIF 452 KB) [file 203_2022_2768_MOESM11_ESM.tif]

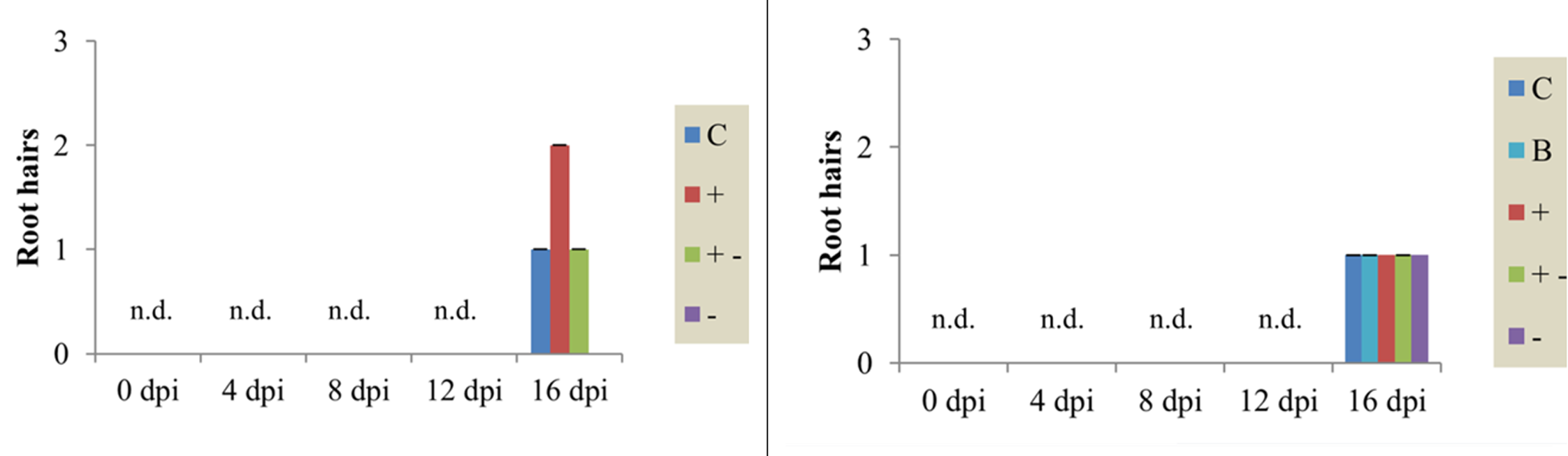

Supplement: Supplementary file 12 — Supplementary file12 (TIF 308 KB) [file 203_2022_2768_MOESM12_ESM.tif]

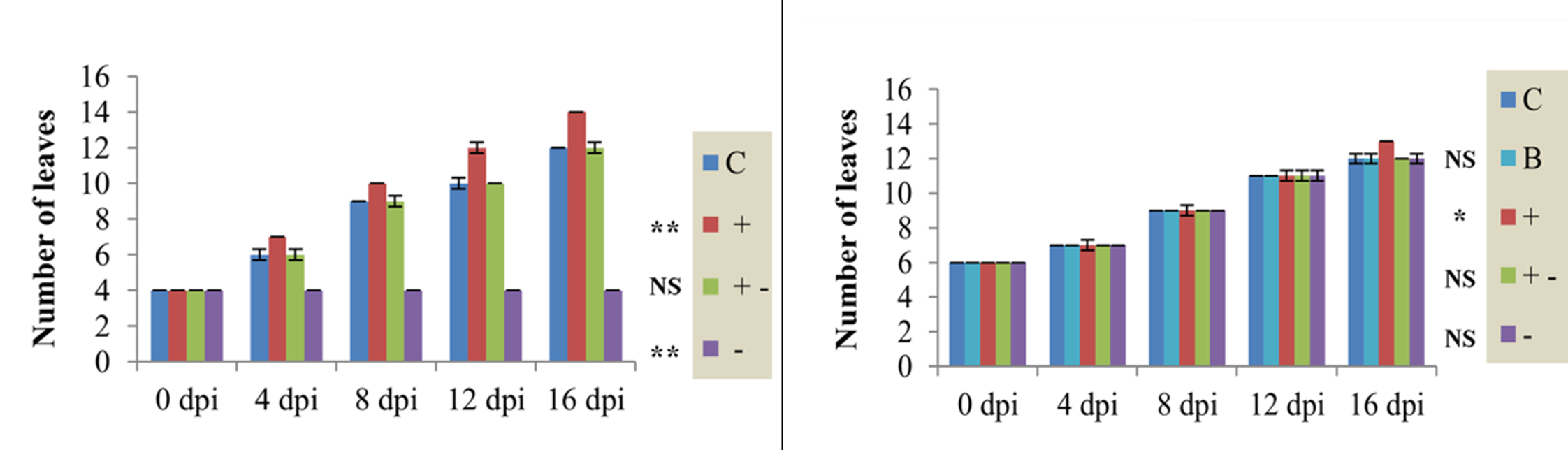

Supplement: Supplementary file 13 — Supplementary file13 (TIF 577 KB) [file 203_2022_2768_MOESM13_ESM.tif]

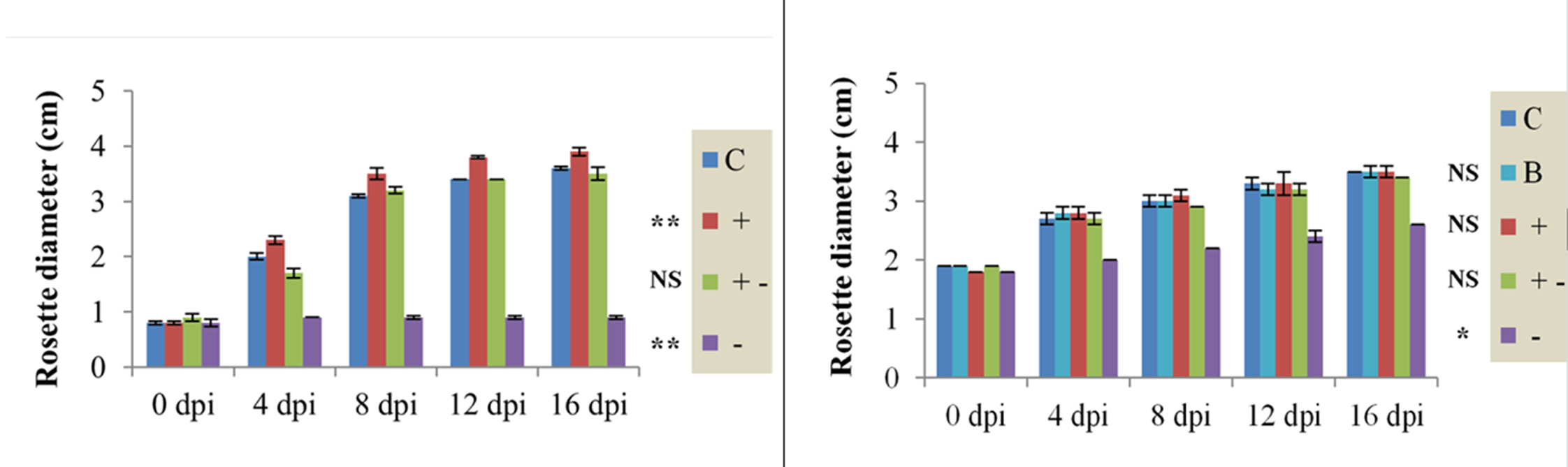

Supplement: Supplementary file 14 — Supplementary file14 (TIF 600 KB) [file 203_2022_2768_MOESM14_ESM.tif]

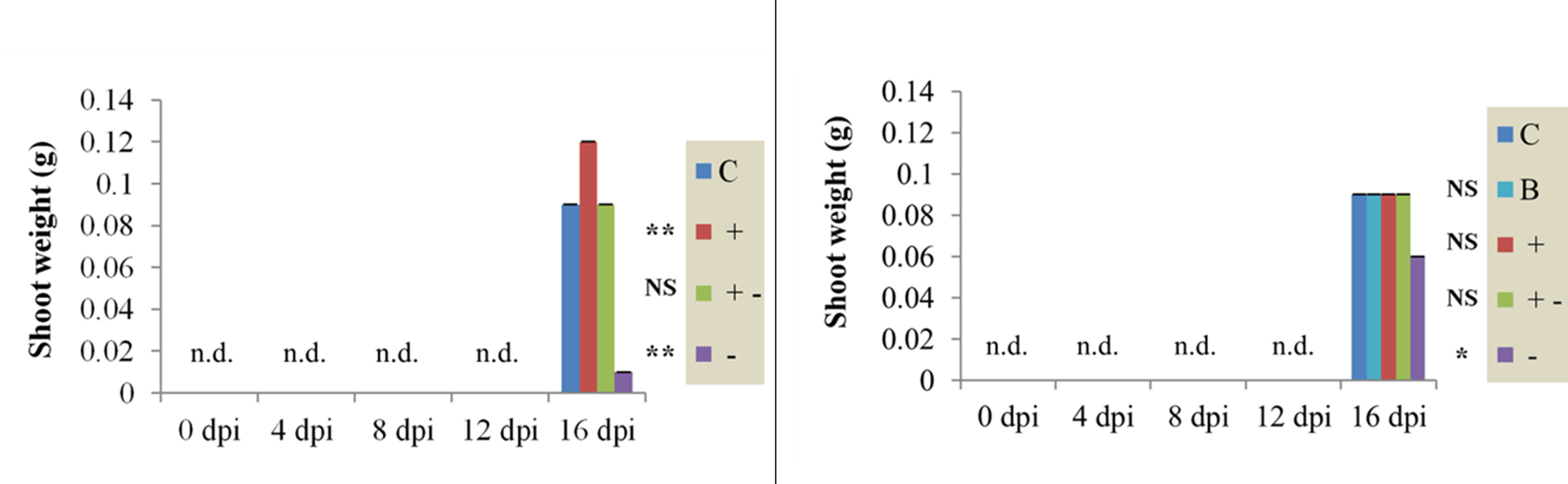

Supplement: Supplementary file 15 — Supplementary file15 (TIF 401 KB) [file 203_2022_2768_MOESM15_ESM.tif]

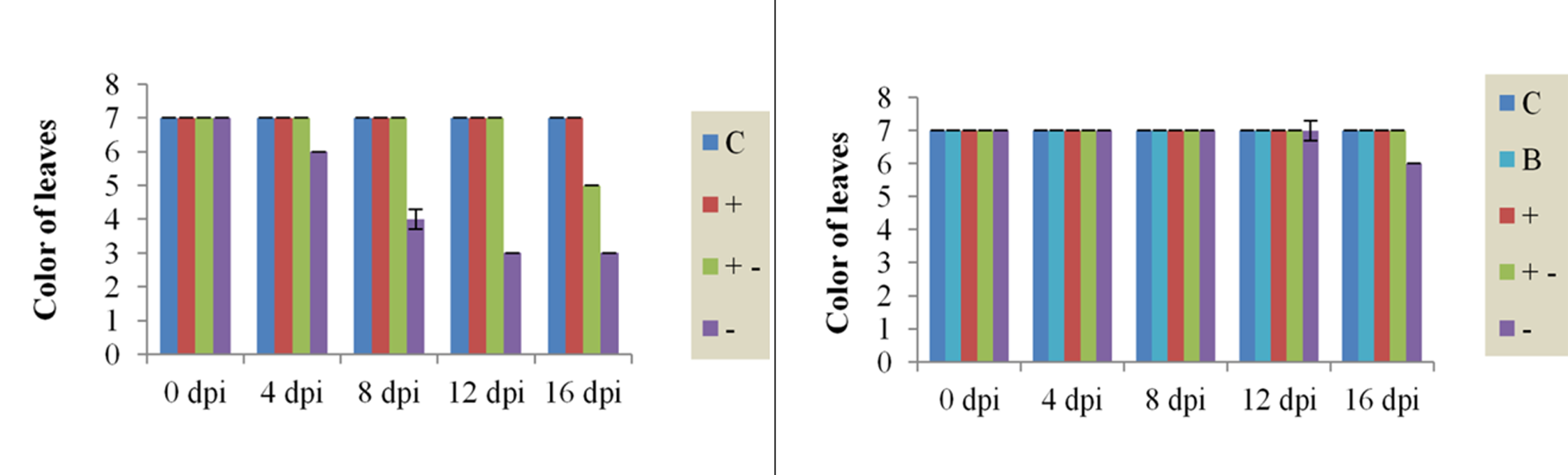

Supplement: Supplementary file 16 — Supplementary file16 (TIF 675 KB) [file 203_2022_2768_MOESM16_ESM.tif]

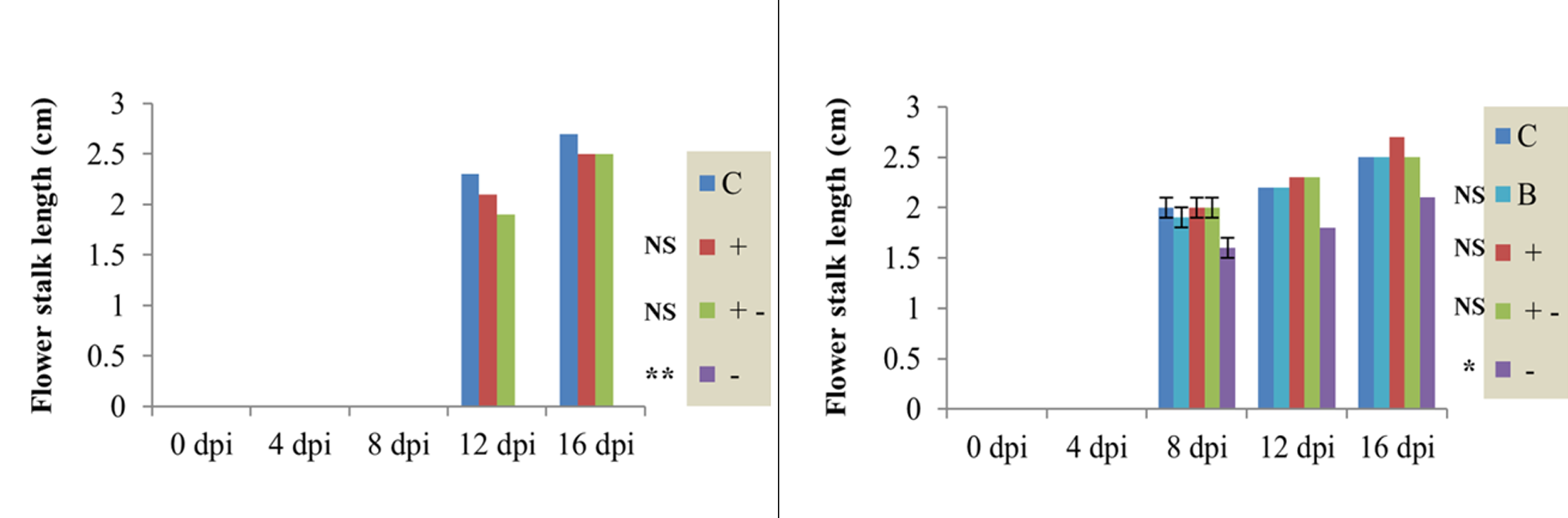

Supplement: Supplementary file 17 — Supplementary file17 (TIF 507 KB) [file 203_2022_2768_MOESM17_ESM.tif]

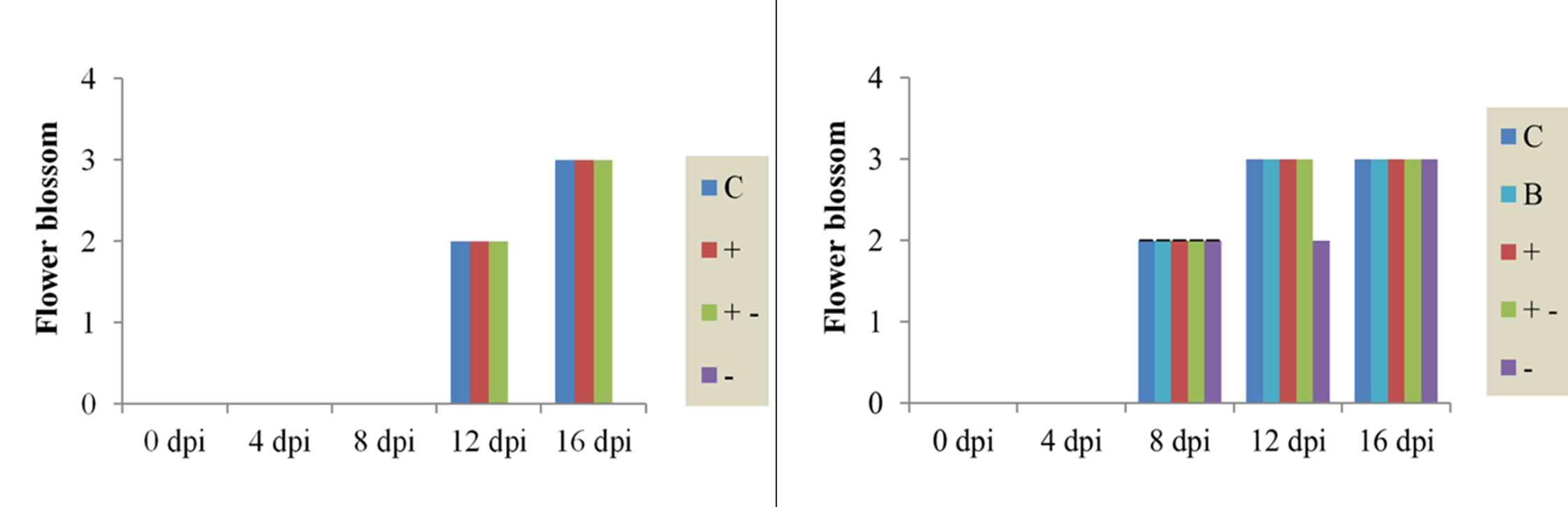

Supplement: Supplementary file 18 — Supplementary file18 (TIF 447 KB) [file 203_2022_2768_MOESM18_ESM.tif]

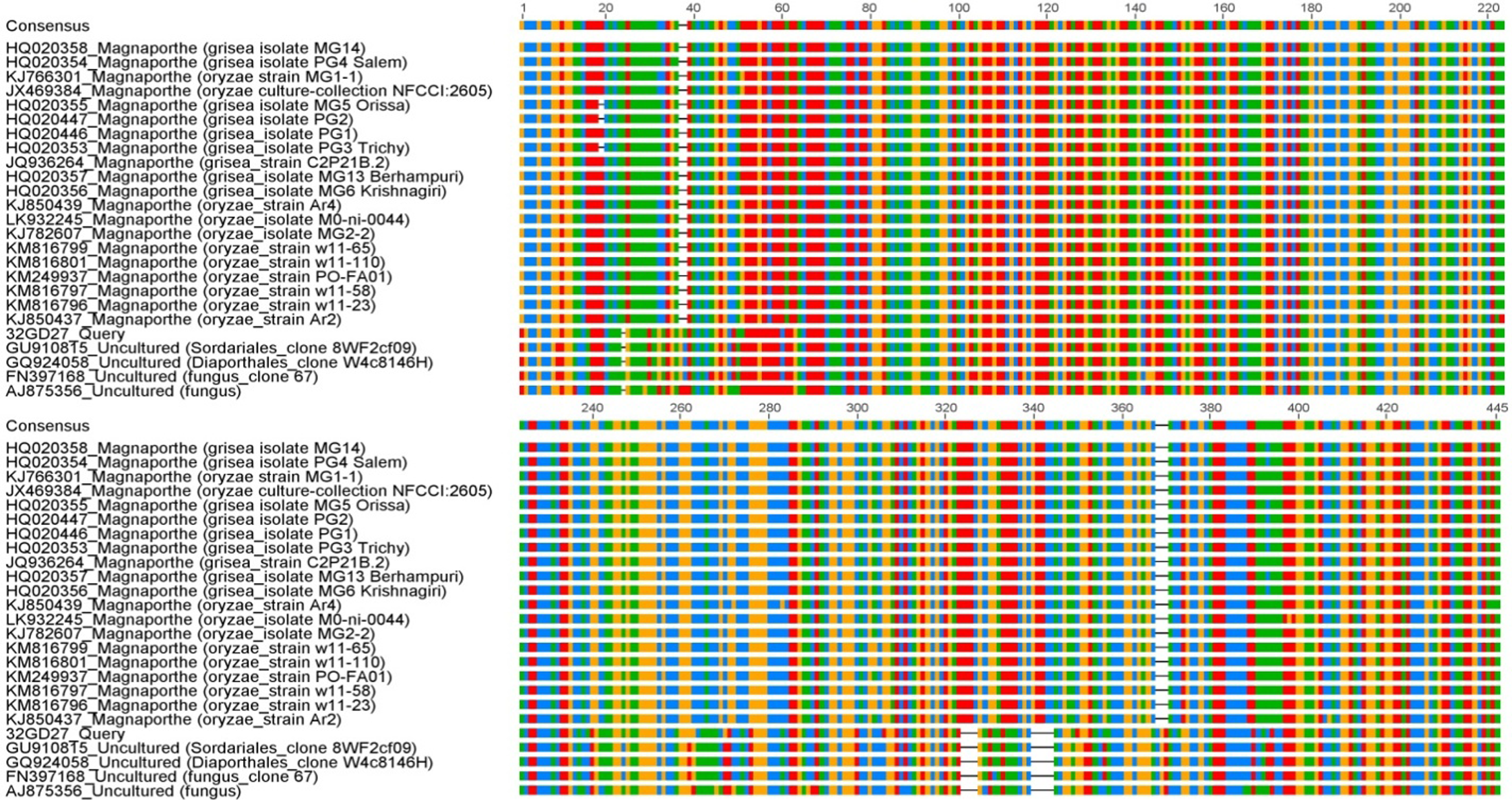

Supplement: Supplementary file 19 — Supplementary file19 (TIF 7814 KB) [file 203_2022_2768_MOESM19_ESM.tif]
